# SOCS2 inhibits hepatoblastoma metastasis via downregulation of the JAK2/STAT5 signal pathway

**DOI:** 10.1038/s41598-023-48591-7

**Published:** 2023-12-09

**Authors:** Yong Lv, Xiaolong Xie, Guoyou Zou, Meng Kong, Jiayin Yang, Jing Chen, Bo Xiang

**Affiliations:** 1 Department of Pediatric Surgery and Laboratory of Pediatric Surgery, West China Hospital, Sichuan University, Chengdu, 610041 China; 2 Department of General Surgery, People’s Hospital of Tibet Autonomous Region, Tibet, 850000 China; 3 Department of Pediatric Surgery, Children’s Hospital Affiliated to Shandong University, Jinan, 250022 China; 4 Liver Transplantation Center, Department of General Surgery, West China Hospital, Sichuan University, Chengdu, 610041 China

**Keywords:** Genetics, Cancer, Metastasis

## Abstract

Metastasis of hepatoblastoma (HB) is a key factor that impairs the prognosis and treatment of children. The suppressor of cytokine signaling 2 (SOCS2) is a classical negative feedback protein that regulates cytokine signal transduction and has been known to be downregulated in several tumor, but the molecular mechanisms of its involvement in HB metastasis are unknown. We found that SOCS2 was a gene down-regulated in hepatoblastoma and associated with HB metastasis through bioinformatics. The qRT-PCR, Western blot and IHC showed that SOCS2 was significantly lower in HB tissues. Clinicopathological correlation analysis revealed that low expression of SOCS2 was significantly correlated with tumor metastasis (P = 0.046) and vascular invasion (P = 0.028), associated with poor prognosis. Overexpression of SOCS2 inhibited the migration and invasion of hepatoblastoma cells, while knockdown of SOCS2 expression promoted these malignant phenotypes. In vivo studies revealed overexpression of SOCS2 inhibited the formation of lung metastasis. Up-regulation of SOCS2 in HB cell inhibited EMT and JAK2/STAT5. Conversely, down-regulation of SOCS2 promoted EMT and JAK2/STAT5. The addition of the JAK2 inhibitor Fedratinib partially reversed the effects of si-SOCS2 on HB cells. SOCS2 may inhibit the migration and invasion of HB cells by inhibiting the JAK2/STAT5 signaling pathway. These results may provide guiding significance for the clinical treatment of HB.

## Introduction

Hepatoblastoma (HB) is a primary malignant liver tumor in children. According to statistics, the incidence of pediatric hepatoblastoma is increasing year by year, posing a serious threat to affected children^1^. HB has an insidious onset and lacks typical clinical symptoms in the early stages, and a large number of children have intrahepatic tumor metastases or distant metastases upon admission, which are resistant to chemotherapy and cannot be completely removed surgically^2^. In addition, children with distant metastases have a poor prognosis, and tumor metastasis is the leading cause of death in HB children^3^. Therefore, conducting thorough research on hepatoblastoma metastasis holds great clinical significance and scientific value.

Bioinformatics has become a popular method for studying complex disease mechanisms using high-throughput sequencing data. Weighted gene co-expression network analysis (WGCNA) is a systems biology approach that has emerged as the most promising method for mining core pivotal genes^4^. This study combines WGCNA and differentially expressed gene analysis to better understand the complex metastatic mechanisms of hepatoblastoma. We found that SOCS2 was closely associated with hepatoblastoma metastasis by WGCNA. SOCS2 is a classical negative regulator of the JAK/STAT signaling pathway, and several studies have shown that inactivation of SOCS proteins has a substantial impact on tumorigenesis and progression^5^. However, the role and function of SOCS2 in different kind of tumors is controversial. Previous studies have found that SOCS2 acts as a tumor suppressor in breast, lung and ovarian cancers and as a pro-carcinogenic factor in chronic myeloid leukemia^6^. Moreover, Li et al. developed a prognostic prediction model for liver cancer based on the Cancer Genome Atlas Project (TCGA) and found that SOCS2 was an independent predictor of prognosis in patients with hepatocellular carcinoma^7^.

Taken together, we found that the SOCS2 may be associated with the malignant biological behavior of tumor. However, no studies of SOCS2 have been reported in hepatoblastoma, and the specific regulatory mechanisms are yet less known. Therefore, the effect of SOCS2 on hepatoblastoma metastasis became the subject of our experiments to be studied.

## Methods

### Weighted gene co-expression network analysis

GSE131329 has comprehensive clinical data, and WGCNA was carried out with this expression profile. WGCNA is a systems biology method for characterizing gene association patterns between different samples, used to identify highly synergistic sets of genes and to screen for candidate biomarker genes or therapeutic targets^8^. We used the WGCNA function package in R software to screen for gene modules associated with disease phenotypes and further analyzed the relationship between these modules and HB tumor samples. We quantify associations of individual genes with our trait of interest (tumor metastasis) by defining Gene Significance (GS) as the correlation between the gene and the trait. For each module, we also defining a quantitative measure of module membership (MM) as the correlation of the module eigengene and the gene expression profile. This allows us to quantify the similarity of all genes on the array to every module^9^. Differentially expressed genes (DEGs) between HB and normal liver were screened from GSE131329 with the “edgeR” package. And significantly changed genes were selected with P value < 0.05 and log_2_|fold change|≥ 1.

### Clinical specimens

The study was approved by the West China Hospital of Sichuan University Biomedical Research Ethics Committee, and informed consent was obtained from all participants or legal guardian. All information regarding the human material was managed using anonymous numerical codes and the experiments were conducted following the Helsinki declaration. We collected specimens from patients with hepatoblastoma admitted to the Department of Pediatric Surgery at West China Hospital of Sichuan University between January 2015 and January 2021, and all children underwent surgery or pathological biopsy in our department. A total of 72 hepatoblastoma children included in this study, and all cases had complete clinical and pathological data. Follow-up visits were conducted by telephone to clarify the living status of the patients and to confirm the time of death and the cause of death. Overall survival (OS) is the time between the diagnosis of a patient and the death of the patient.

### Cell lines and cell culture

The human hepatoblastoma cell lines Huh6, HepG2, and normal liver cells LO2 were purchased from the cell bank (Chinese Academy of Sciences). The process of cell culture and passaging involves changing the medium every 1–2 days. Remove the old culture medium (DMEM supplemented with 10% FBS) and add 1.5 ml trypsin to the culture vessel, after 2 min, add culture medium to terminate digestion. Transfer the cells to a 15 ml centrifuge tube, centrifuge at 1000 rpm for 5 min at room temperature, and discard the supernatant. Add 2 ml culture medium to the centrifuge tube, gently blow and mix the cell pellet, and then collect cell suspension. Add the suspension to a culture vessel and place in a 37℃, 5% CO2 cell incubator for further culture.

### Ectopic SOCS2 overexpression and knockdown of hepatoblastoma cells

To establish an overexpression plasmid of SOCS2, the CDS sequence of SOCS2 was searched for in the Pubmed Nucleotide database. The restriction enzyme sites were analyzed and primers with enzyme sites were designed. The target gene fragment was amplified using cDNA as a template, processed with restriction enzyme sites, linearized pCS2 vector, ligated with the fragment, transformed DH5α competent cells, identified positive clones by PCR and sequencing. The si-RNA used in this project is purchased from Tsingke Biotechnology Co., Ltd. The transfections were performed by Lipofectamine 8000 (Beyotime, China).

### Quantitative reverse transcription PCR

Total RNA is extracted from tissue or cell using Trizol reagent. Then Reverse transcription of RNA was Carried out with reverse transcription primer and reverse transcriptase. Quantitative PCR is set up using Taq Master Mix (Novoprotein Scientific Inc.), primers, reverse transcription product, and RNase Free water. PCR amplification was performed at 95 °C for 5 min, followed with cycling conditions of 40 cycles at 95 °C for 15 s, 60 °C for 10 s, and 72 °C for 20 s in a real time-PCR system with SYBR green.

### Immunohistochemistry

The paraffin sections are deparaffinized and rehydrated before restoring the antigen using citrate buffer at high temperature. Endogenous peroxidase activity is eliminated with H_2_O_2_, and non-specific antigen is eliminated with goat serum. Primary antibody working solution is added and incubated overnight, followed by secondary antibody and DAB chromogenic solution. The results are determined based on the proportion and intensity of positive cells, with a total score of 0–6. The expression level is classified as negative (0 point), weakly positive (1–2 points), moderately positive(3 points), or strongly positive (4–6 points), with low expression defined as a total score of 0–2 and high expression defined as a total score of 3–6.

### Western blot

Extracting and preparing protein from tissue or cells. Placing the 10% SDS-PAGE in the electrophoresis apparatus, and loading the samples into the wells. Electrophoresis is run at a constant voltage to separate the proteins. Then transferred protein from the gel onto a PVDF membrane. The next step is to block the membrane with 5% skimmed milk, incubating with primary and secondary antibodies, and washing the PVDF membrane. The final step is to detect the target protein using a detection system and analyzing the results using Image J software.

### Wound-healing assay

The procedure for scratch healing involves marking a 6-well plate, seeding HB cells, transfecting si-RNA or plasmid and continue to incubate HB cells, scratching the wells with a 200 μL pipette tip. Washing and imaging the cells after 48 h, and calculating the healing rate using Image J software.

### Transwell assays

The cells are digested and suspended in serum-free medium to adjust the concentration to 2 × 10^5^/mL. The lower chamber of a 24-well plate is filled with medium containing 10% serum, while the upper chamber is filled with cell suspension. After a period of continued incubation, then chambers are fixed with methanol and stained with Giemsa solution. After rinsing with water, the cells on the upper surface of the chambers are carefully removed.

### In vivo studies

The study is reported in accordance with ARRIVE guidelines. All procedures were performed in accordance with guidelines for animal research and approved by the west china hospital of Sichuan University Animal Ethics Committee (Approval Number: 20230106004). Total 20 nude mice (4–5 weeks old, female) were purchased from Beijing Hfk Biosicence Co., Ltd. and divided into two groups randomly (n = 10 per group). Animals were maintained in the specific pathogen-free facility with standard 12-h light/dark cycles, allowed chow and water ad libitum, and euthanized humanely in their home cages with CO_2_ followed by cervical dislocation. Huh6 cells transfected with SOCS2 overexpression Lentivirus (LV-SOCS2-Huh6) or Ctrl-Lentivirus (LV-NC-Huh6). And the EGFP-labeled Lentivirus was purchased from GENECHEM Co., Ltd. Then Huh6 cells (2 × 10^5^/200 μL) infected with lentiviruses were injected into the tail vein of female BALB/c-nude mice. After 4 weeks, the mice were euthanized and the lungs were removed for taking pictures. The IVIS® Lumina III in vivo imaging system to monitor for lung metastasis, imaged ex vivo. The EGFP luminescence was quantified as average radiance using the Living Image software version 4.0 and the region of interest (ROI) tool. After fluorescence imaging is completed, lungs were fixed in 10% formalin for HE staining.

### Statistical analysis

GraphPad Prism9.0 and SPSS25.0 were used to perform statistical analysis. Mean ± standard deviation was used to represent quantitative data, and independent sample t-test or ANOVA was used to compare groups. Non-normally distributed data was represented using median. The correlation between SOCS2 expression and HB clinical pathological characteristics was analyzed using chi-square test, with P < 0.05 indicating statistical significance.

### Ethics approval and consent to participate

The study was approved by the West China Hospital of Sichuan University Biomedical Research Ethics Committee (No. 1085), and informed consent was obtained from all participants or legal guardian. All information regarding the human material was managed using anonymous numerical codes and the experiments were conducted following the Helsinki declaration.

## Results

### Identification of the hub hepatoblastoma metastasis-related genes by WGCNA

**Figure 1:**
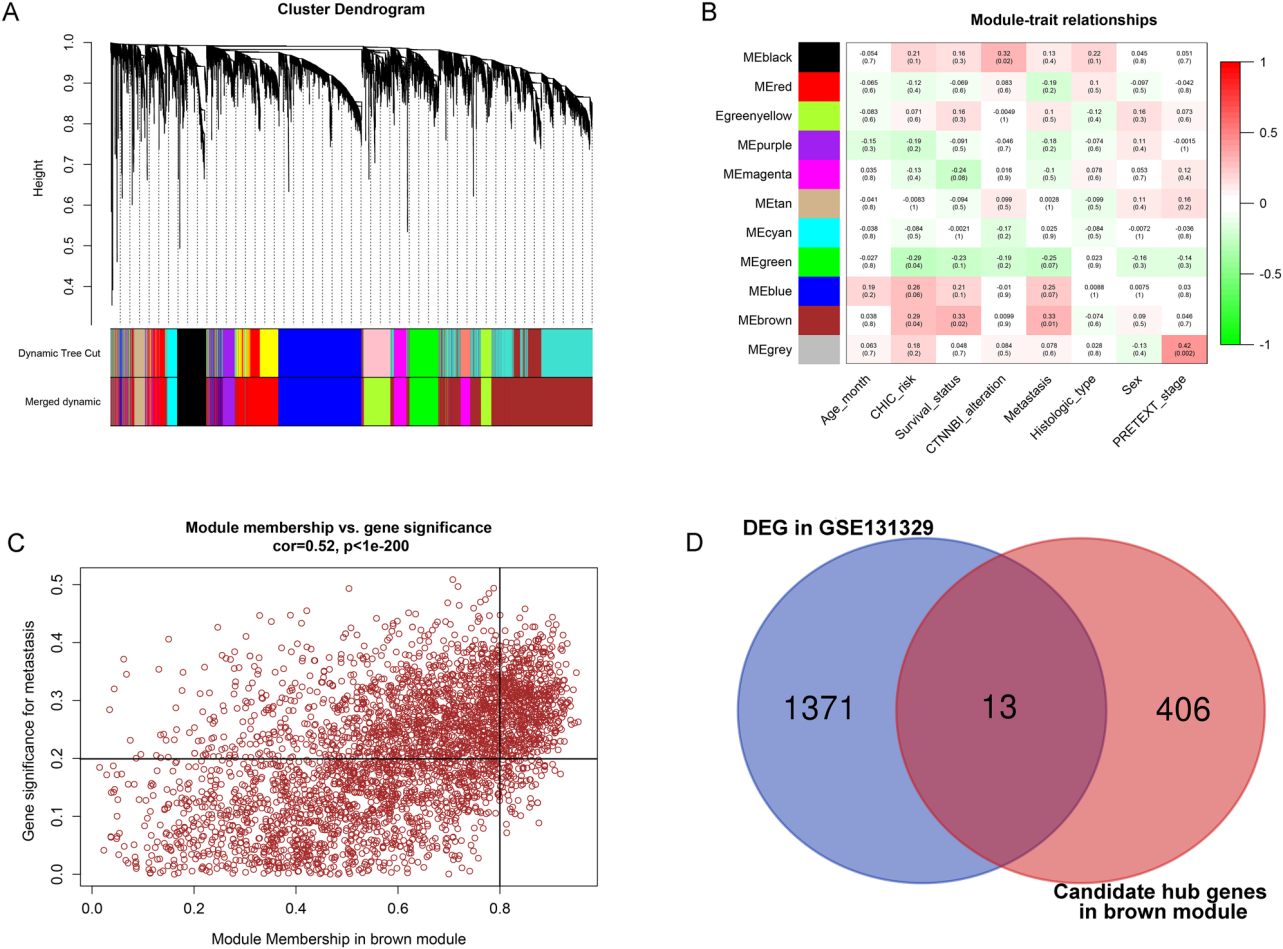
Weighted gene co-expression network analysis to identify hepatoblastoma metastasis related genes. (**A**) The cluster dendrogram of genes. Genes that could not be clustered into one of these modules were assigned to the gray module. Every gene represents a line in the hierarchical cluster. (**B**) Heatmap of the module-trait relationships. (**C**) Scatterplots of gene significance (GS) versus module membership (MM) in brown modules. (**D**) Venn diagrams of DEGs and co-expressed genes of GSE131329 dataset.

The expression profiles of genes in GSE131329 were analyzed using the WGCNA package in R software. We obtained 11 different gene modules (Fig. 1A). The brown module was the gene module most associated with HB metastasis (Fig. 1B). The brown gene module contains 3639 genes, of which a total of 419 genes with high similarity and significance (Module Membership > 0.8 and Gene Significance > 0.2) were screened as candidate hub genes (Fig. 1C). And then these genes were intersected with the differential expressed genes in hepatoblastoma, and finally 13 mRNAs were obtained (Fig. 1D). Because SOCS2 is both associated with hepatoblastoma metastasis and has the largest differential expression fold (|logFC|= 1.87), we selected SOCS2 as the molecule for subsequent experimental analysis in this study (Table 1).

**Table 1:** The hub hepatoblastoma metastasis-related genes.

mRNA	logFC	GS metastasis	MM brown
SOCS2	− 1.87334877	0.444031713	0.902965478
ERCC2	1.10109654	0.264548437	0.897494982
NRSN2	1.26354292	0.311453279	0.877873758
SPC24	1.42266372	0.354176817	0.868872464
GLMP	1.19052892	0.261994628	0.841013447
EHMT2	1.31973298	0.221036295	0.893307911
TP53	1.14023764	0.246125749	0.873288247
HMGA1	1.01327412	0.331682212	0.800994717
NPM3	1.08892011	0.333428331	0.837285308
EDIL3	1.84189458	0.285393253	0.801461928
NREP	1.75462558	0.247086922	0.825266711
SLC31A1	− 1.13012596	0.204139803	0.871592592
SATB1	− 1.26706906	0.222952383	0.872608897

*MM* module membership, *GS* gene significance.

### Low expression of SOCS2 in hepatoblastoma

**Figure 2:**
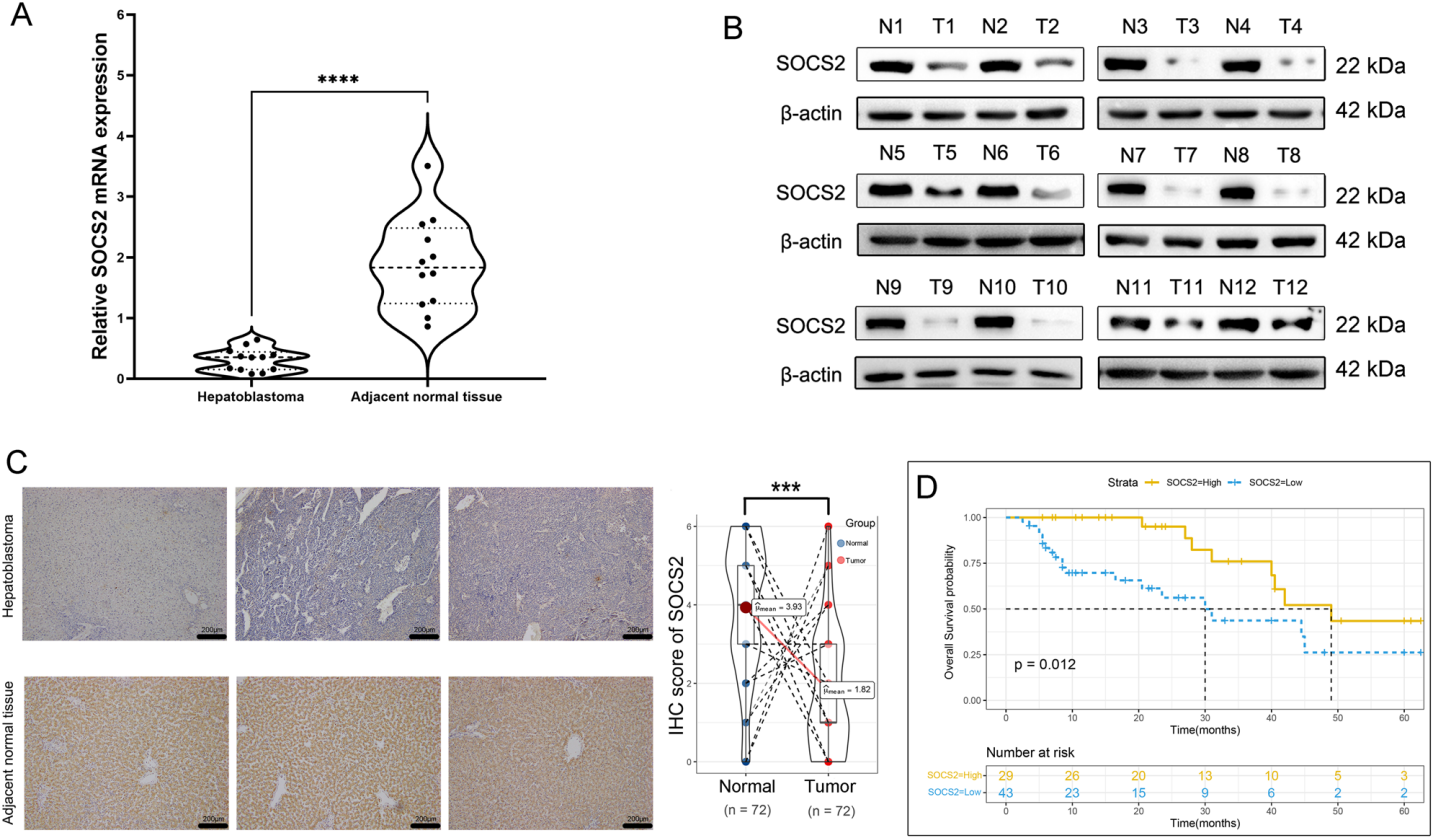
SOCS2 expression is down-regulated in hepatoblastoma. (**A**) The SOCS2 mRNA expression in 12 paired hepatoblastoma patients quantified by qRT-PCR. Analysis was performed using the paired t test and ****p < 0.0001. (**B**) The SOCS2 protein expression was detected by western blotting in 12 pairs of hepatoblastoma tissues (T) and adjacent normal tissues (N). (**C**) IHC analysis of SOCS2 expression in hepatoblastoma. Representative images were shown (scale bar 200 μm). The violin plot described the IHC score of SOCS2 in hepatoblastoma. Analysis was performed using the paired t test and ***p < 0.001. (**D**) Kaplan–Meier curves of overall survival (OS) in hepatoblastoma stratified by SOCS2 expression. Analysis was performed using the Log-rank test and p = 0.012.

We used qRT-PCR to analyze the mRNA levels of SOCS2 in tumor tissues and paired paraneoplastic liver tissues of 12 children with hepatoblastoma in our hospital, and we found that the mRNA levels of SOCS2 in HB tissues were significantly lower than those in paraneoplastic liver tissues (Fig. 2A). Western blot also showed that SOCS2 protein levels were significantly lower in HB tissue than in paraneoplastic liver tissue (Fig. 2B). Then we performed immunohistochemistry (IHC), among 72 hepatoblastoma samples collected in our hospital, we found that SOCS2 showed low expression in 43 (59.72%) hepatoblastoma tissues and only 16 (22.22%) in paired paraneoplastic liver tissues, and comparing the IHC scores, we found that the IHC scores of SOCS2 in paraneoplastic liver tissues were significantly higher than those of tumor tissues (Fig. 2C). Moreover, from clinical data we found that SOCS2 expression was correlated with tumor metastasis and vascular invasion (Table 2). The low SOCS2 expression group had a significantly lower survival rate compared to the high expression group, as demonstrated by the Kaplan–Meier survival curve (Fig. 2D).

**Table 2:** Correlation between SOCS2 and clinicopathological characteristics of HB.

Character	High	Low	Overall	P
	(N = 29)	(N = 43)	(N = 72)	value
Gender				
Female	12 (41.4%)	16 (37.2%)	28 (38.9%)	
Male	17 (58.6%)	27 (62.8%)	44 (61.1%)	0.722
Age				
> 57 months	16 (55.2%)	24 (55.8%)	40 (55.6%)	
≤ 57 months	13 (44.8%)	19 (44.2%)	32 (44.4%)	0.957
Tumor size				
< 10 cm	16 (55.2%)	22 (51.2%)	38 (52.8%)	
≥ 10 cm	13 (44.8%)	21 (48.8%)	34 (47.2%)	0.738
Histology type				
Epithelial	20 (69.0%)	30 (69.8%)	50 (69.4%)	
Mixed	9 (31.0%)	13 (30.2%)	22 (30.6%)	0.942
Vascular invasion				
Absent	24 (82.8%)	25 (58.1%)	49 (68.1%)	
Present	5 (17.2%)	18 (41.9%)	23 (31.9%)	0.028
Metastasis				
Absent	25 (86.2%)	28 (65.1%)	53 (73.6%)	
Present	4 (13.8%)	15 (34.9%)	19 (26.4%)	0.046
PRETEXT stage				
I_II	16 (55.2%)	22 (51.2%)	38 (52.8%)	
III_IV	13 (44.8%)	21 (48.8%)	34 (47.2%)	0.738
COG stage				
I_II	17 (58.6%)	29 (67.4%)	46 (63.9%)	
III_IV	12 (41.4%)	14 (32.6%)	26 (36.1%)	0.445
AFP				
< 100 ng/mL	1 (3.4%)	3 (7.0%)	4 (5.6%)	
≥ 100 ng/mL	28 (96.6%)	40 (93.0%)	68 (94.4%)	0.521

### SOCS2 inhibits migration and invasion of hepatoblastoma cells

**Figure 3:**
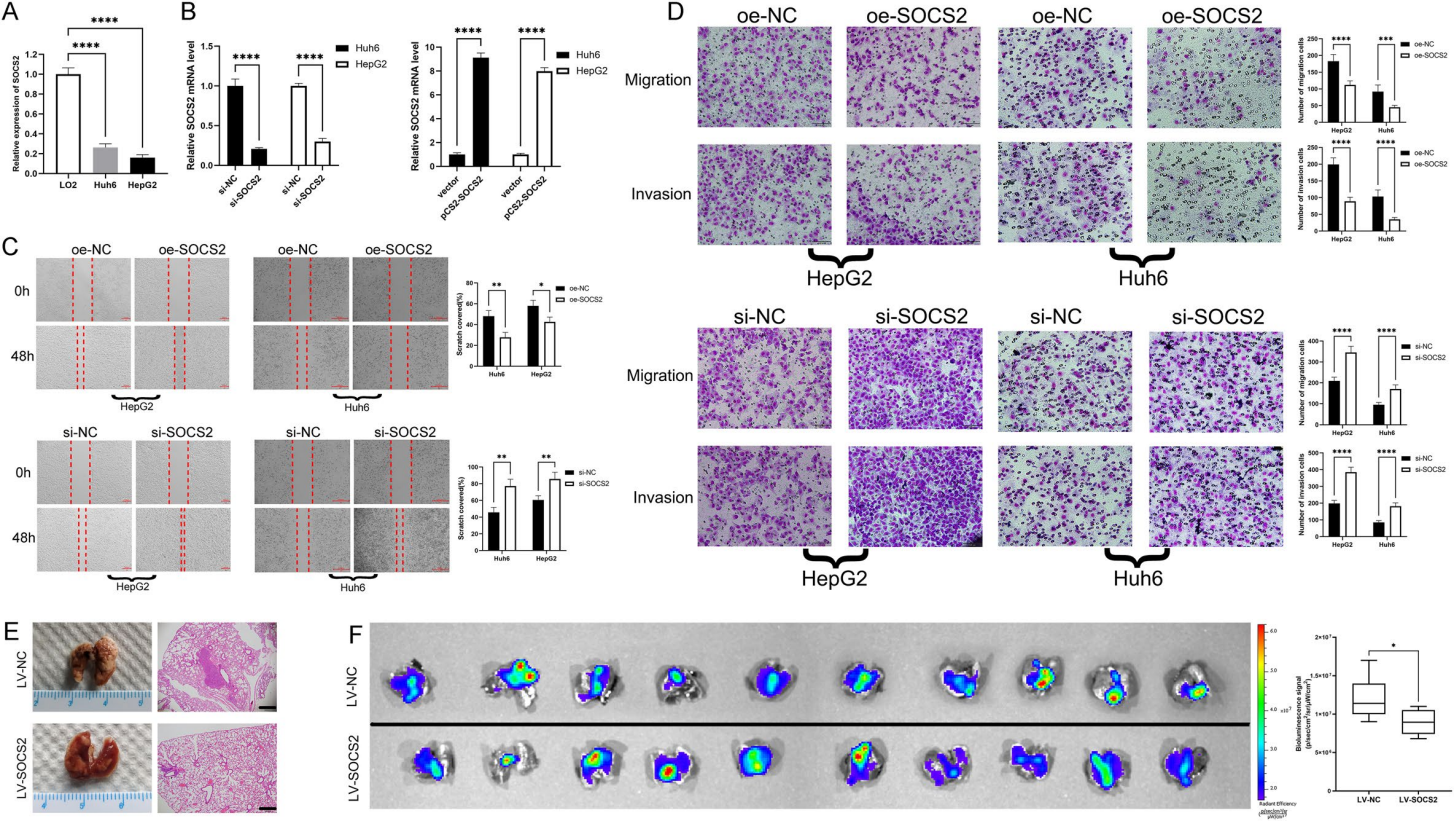
SOCS2 inhibited migration and invasion of hepatoblastoma cells. (**A**) Endogenous SOCS2 mRNA expression status was compared between LO2 and hepatoblastoma cells (Huh6 and HepG2) by qRT-PCR. (**B**) qRT-PCR analysis of SOCS2 mRNA levels in SOCS2 overexpression or knockdown in hepatoblastoma cells. (**C**) Wound-healing assay was used to detect cell migration ability of SOCS2 overexpressed or SOCS2 knockdown hepatoblastoma cells (left: representative images; right: quantitative analyses). (**D**) Transwell assays showed the effect of SOCS2 overexpression or SOCS2 knockdown on hepatoblastoma cell migration and invasion (left: representative images; right: quantitative analyses). (**E**) SOCS2 overexpression cells led to a smaller lung metastatic area compared to control cells. (left: the representative ex vivo lungs images; right: the representative HE images, scale bars represent 500 μm). (**F**) Average bioluminescence signal measured in the lungs of control group was higher than in lungs of SOCS2 overexpression group. (left: Bioluminescence images; right: quantitative analyses of bioluminescence). Analysis was performed using the ANOVA in (**A**–**D**) and the unpaired t test in (**F**), *p < 0.05, **p < 0.01, ***p < 0.001 and ****p < 0.0001.

To explore the underlying biological function of SOCS2 in hepatoblastoma, we first detected the mRNA expression levels of SOCS2 in HB cells (Huh6, HepG2) and hepatocytes LO2 by qRT-PCR, and the results showed that SOCS2 was significantly lower in HB cells than in LO2 cell line (Fig. 3A). We used siRNA and overexpression plasmids to knock down or elevate SOCS2 in HB cells, respectively; the qRT-PCR showed high transfection efficiency (Fig. 3B), allowing for subsequent biological function studies. Cell scratch assay showed that overexpression of SOCS2 inhibited the migratory healing ability of HB cells (Fig. 3C), while the migratory healing ability of HB cells was significantly enhanced after knockdown of SOCS2 (Fig. 3D). Transwell experiment showed that the migration and invasion ability of HB cells overexpressing SOCS2 was significantly decreased, while significantly increased with SOCS2 knockdown. The above results suggest that SOCS2 has an inhibitory effect on the migration and invasive ability of hepatoblastoma cells. We infected Huh6 cells with EGFP labeled lentivirus to construct HB cell lines stably overexpressing SOCS2 (LV-SOCS2-Huh6). Further investigation in vivo indicated that compared to the control group, the LV-SOCS2-Huh6 group nude mice had a smaller area of metastatic lesions in the lung tissue (Fig. 3E). At the end of the 4-week study period, the average bioluminescence signal measured in the ex vivo lungs of control group mice were over 1.33 times higher than in the LV-SOCS2-Huh6 group (Fig. 3F). The results showed that overexpression of SOCS2 impaired formation of hepatoblastoma lung metastasis in vivo.

### GSEA identifies the signal pathway associated with SOCS2 in hepatoblastoma

**Figure 4:**
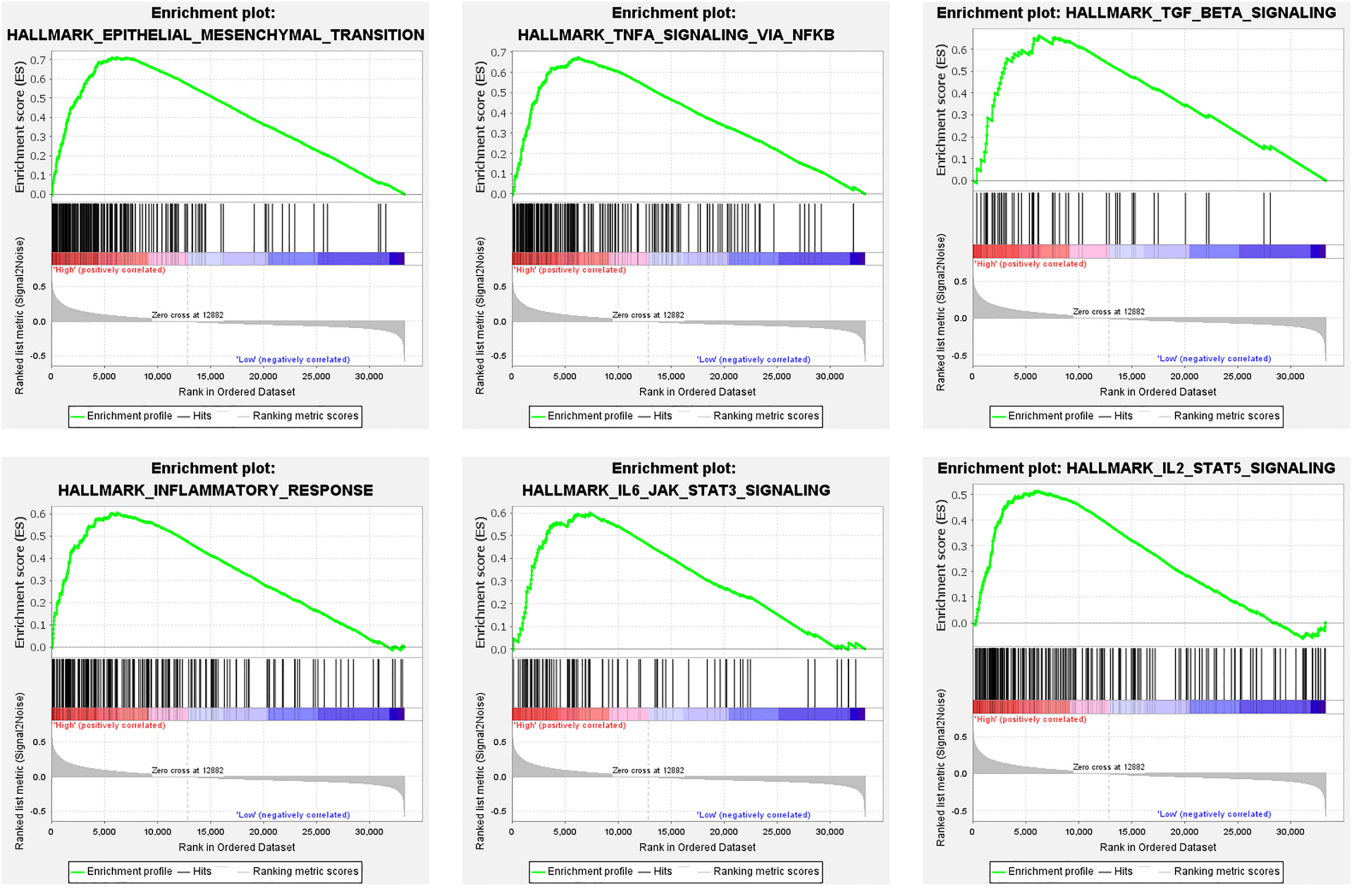
Gene set enrichment analysis between high and low SOCS2 expression using the GSE131329 hepatoblastoma gene expression data.

To further explore the downstream mechanisms of SOCS2 in inhibiting HB metastasis, we performed GSEA analysis of hepatoblastoma gene expression profiles in GSE131329 to identify the biological processes and signaling pathways affected by SOCS2. GSEA suggests that the biological process with the highest enrichment score is epithelial mesenchymal transition, followed by regulation of immune-inflammation related signaling pathways, two of which are closely related to the JAK/STAT pathway, namely IL2-STAT5 and IL6-JAK-STAT3 signaling pathways (Fig. 4). Previous studies have shown that aberrantly activated JAK2/STAT5 can promote EMT^10^. While epithelial mesenchymal transition is a key step in the metastasis process. We will next explore the effect of SOCS2 on the JAK2/STAT5 signaling pathway in HB cells.

### SOCS2 inhibits the JAK2/STAT5 signal pathway and EMT

**Figure 5:**
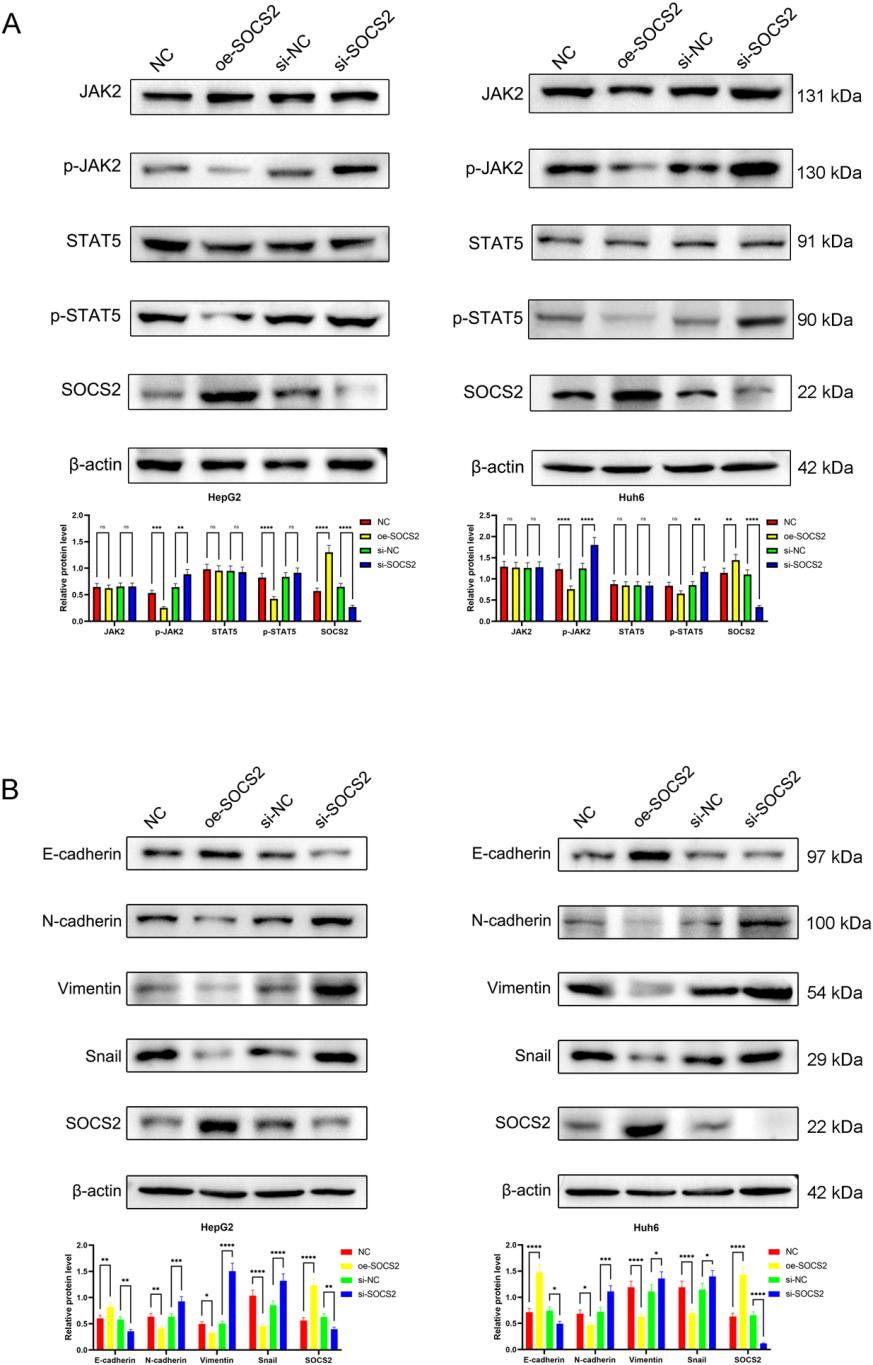
SOCS2 overexpression inhibits JAK2/STAT5 signal and EMT of hepatoblastoma cells. (**A**) Western blotting analysis of JAK2/STAT5 signaling key components (p-JAK2, p-STAT5, JAK2, and STAT5) in hepatoblastoma cells after SOCS2 overexpression or SOCS2 knockdown. (**B**) EMT markers of E-cadherin, N-cadherin, Vimentin, and Snail protein expression levels in SOCS2-overexpression or SOCS2-knockdown hepatoblastoma cells were detected by western blotting. Analysis was performed using the ANOVA and ns denotes p > 0.05, *p < 0.05, **p < 0.01, ***p < 0.001 and ****p < 0.0001.

We altered SOCS2 expression in HB cells, and western blot results showed that overexpression of SOCS2 inhibited phosphorylation of JAK2 and STAT5, while knockdown of SOCS2 promoted the phosphorylation of p-JAK2 and p-STAT5, while total protein levels of JAK2 and STAT5 were unchanged (Fig. 5A). In HB cells, SOCS2 overexpression significantly increased the expression of epithelial marker (E-cadherin) and decreased the expression of mesenchymal markers (N-cadherin, Vimentin and Snail); in contrast, silencing SOCS2 expression in HB cells greatly inhibited the expression of E-cadherin while increased the expression of N-cadherin, Vimentin and Snail (Fig. 5B).

### SOCS2 regulates EMT in hepatoblastoma through JAK2/STAT5 signal pathway

**Figure 6:**
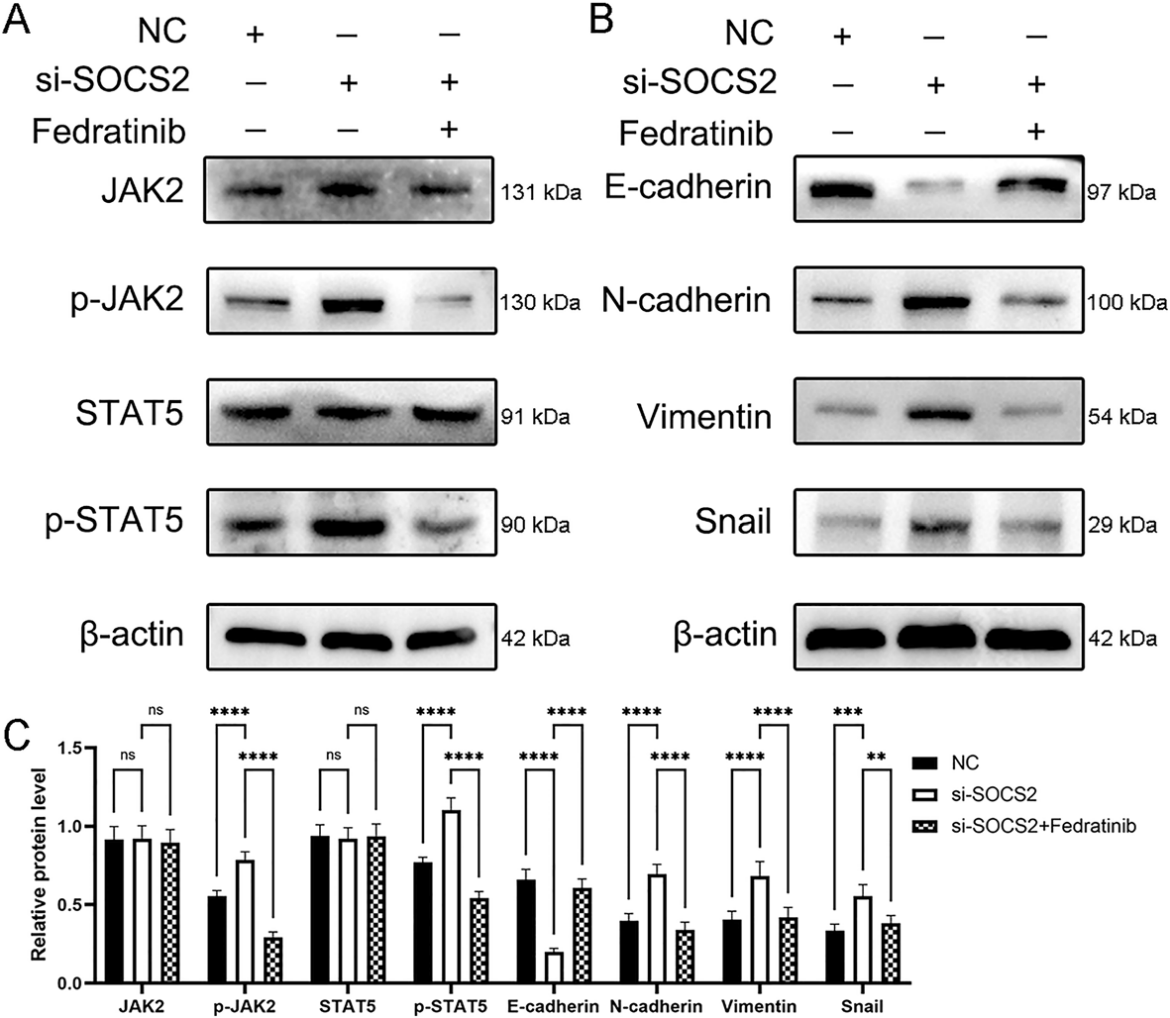
SOCS2-knockdown Huh6 were treated with JAK2 inhibitor Fedratinib. (**A**) After treated for 24 h, as indicated, p-JAK2, p-STAT5, JAK2, and STAT5 protein expression levels were detected by western blotting. (**B**) After treated for 24 h, as indicated, E-cadherin, N-cadherin, Vimentin, and Snail protein expression levels were detected by western blotting. (**C**) The histogram represents a densitometric analysis performed to quantify the relative intensity of bands detected by western blotting, with β-actin as the loading control. Analysis was performed using the ANOVA and ns denotes p > 0.05, *p < 0.05, **p < 0.01, ***p < 0.001 and ****p < 0.0001.

To further determine whether SOCS2 affects EMT of HB cells via JAK2/STAT5, we applied the JAK2 inhibitor Fedratinib (TG101348) to treat Huh6 cells with knockdown of SOCS2. Rescue experiments showed that the addition of Fedratinib to HB cells with knockdown of SOCS2 was able to partially rescue the effect of siRNA on the JAK2/STAT5 signaling pathway in HB cells, mainly manifested by the decrease of p-JAK2 and p-STAT5 protein levels in HB cells, while the total protein levels of JAK2 and STAT5 remained unchanged (Fig. 6A). Fedratinib was also able to partially rescue the effects of siRNA on EMT in HB cells, as evidenced by increased protein levels of epithelial markers (E-cadherin) and decreased protein levels of mesenchymal markers (N-cadherin, Vimentin and Snail) in HB cells (Fig. 6B). These data demonstrate the importance of SOCS2 in suppressing EMT in HB cells, and suggest that SOCS2 may inhibit EMT in HB cells through the JAK2/STAT5 signaling pathway.

### SOCS2 regulates the migration and invasion of hepatoblastoma through JAK2/STAT5 signaling pathway

**Figure 7:**
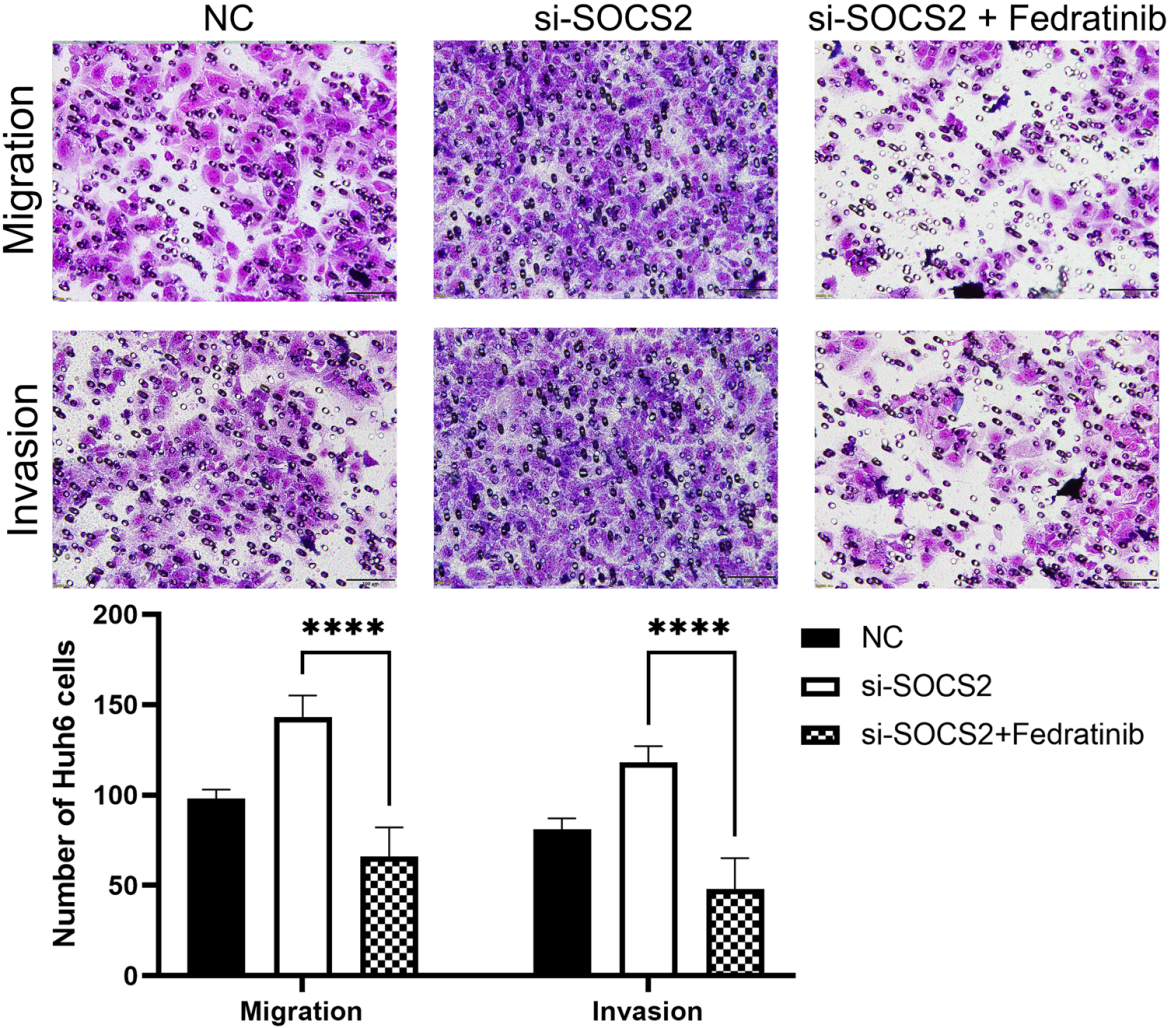
Fedratinib blocks si-SOCS2-mediated cell invasion and migration in Huh6 cell (up panel: representative images; lower panel: quantitative analyses. Analysis was performed using the ANOVA and ****p < 0.0001).

Next, we assessed the role of the SOCS2/JAK2/STAT5 axis in migration and invasion of HB cells. Through Transwell experiment, we found that si-SOCS2 can promote the migration and invasion of HB cells, while Federatinib can reverse the effect of si-SOCS2 on the migration and invasion of hepatoblastoma (Fig. 7). These results suggest that SOCS2 may inhibit the migration and invasion of HB cells through inhibiting the JAK2/STAT5 signaling pathway.

## Discussion

Pulmonary metastasis from hepatoblastoma is a major risk factor in the poor prognosis of children with HB^11^. The molecular mechanism and clinical treatment of pulmonary metastasis from hepatoblastoma remains a thorny issue, requiring extensive in-depth research to improve the prognosis of such patients. In this study, we obtained RNA-seq and clinical data of hepatoblastoma from the GEO database and used WGCNA combined with differential gene expression analysis to find that SOCS2 is associated with hepatoblastoma metastasis. Then we found that SOCS2 expression was significantly reduced in hepatoblastoma both at the mRNA and protein levels by qRT-PCR and Western blot. SOCS2 has been previously reported to act as a tumor suppressor, significantly downregulating in many types of solid tumors^6^. Downregulation of SOCS2 is significantly associated with TNM stage of hepatocellular liver cancer and appears to be a prognostic marker^12^. Additionally, the literature reports that low expression of SOCS2 in primary prostate cancer tissues is associated with an increased incidence of metastasis after radical prostatectomy^13^. Consistently, we found that SOCS2 was associated with hepatoblastoma vascular invasion and tumor metastasis by immunohistochemistry. We explored the expression of SOCS2 in hepatoblastoma for the first time and proposed that SOCS2 may be one of the important regulators of hepatoblastoma metastasis.

We found that SOCS2 inhibits hepatoblastoma invasion and metastasis through cellular experiments. In recent years, researchers have reported the role of SOCS2 in different diseases; however, the role and function of SOCS2 in tumors is controversial. Previous studies have found that SOCS2 plays an anti-tumor role in breast cancer and colon cancer, while it acts as a pro-cancer factor in chronic myeloid leukemia. Low expression of SOCS2 leads to dysregulation of the breast cancer cell cycle and promotes breast cancer cell growth^14^. In colorectal cancer, SOCS2 inhibits Caco-2 cell proliferation and promotes cell differentiation^15^. In contrast, SOCS2 is overexpressed in primary cells of patients with CML, and these patients are in advanced stages of the disease. Our study describes the possible biological role of SOCS2 in hepatoblastoma metastasis and provides new evidence for the treatment of children with hepatoblastoma.

Our study found that SOCS2 inhibits the migration and invasion of hepatoblastoma cells by inhibiting the JAK2/STAT5 signaling pathway. Federatinib is a kinase inhibitor that specifically targets JAK2, among other kinases. However, it does not directly affect the expression of JAK2. By blocking the kinase activity of JAK2, federatinib can disrupt the phosphorylation and activation of downstream targets, including STAT5, leading to the modulation of various cellular processes regulated by the JAK2/STAT5 signaling pathway^16^. Our rescue experiments showed that Fedratinib was able to partially rescue the effect of siRNA on the JAK2/STAT5 signaling pathway in HB cells. JAK/STAT signaling pathway can regulate processes such as cell proliferation, differentiation, migration, and apoptosis. Abnormal and excessive activation of JAK/STAT can lead to uncontrolled cell proliferation, promote tumor cell invasion and metastasis^17^. The JAK2/STAT5 signaling pathway plays a crucial role in the development and functioning of various organs in the human and mouse, including the liver, and the regulation of SOCS2 is crucial for hepatocytes to maintain normal physiological status^18^. The JAK2 protein is responsible for transmitting signals from cytokines to the STAT5 protein, which then activates the expression of genes that regulate cell growth and differentiation. Disruption of JAK2/STAT5 signaling pathways can lead to abnormal liver function, inflammation, and even liver tumor^19^. The JAK2/STAT5 signaling pathway is activated in various types of tumors and is considered a promising drug target for tumor treatment^20^. Targeting JAK2/STAT5 signaling pathway might serve as a potential therapy strategy for metastatic hepatoblastoma.

Epithelial mesenchymal transition is a key process in the development of tumor metastasis. STAT5 protein, as an important transcription factor, plays an important role in the induction of EMT, and activation of STAT5 can promote the occurrence of EMT in tumor cells^21^. The characteristics of EMT include the downregulation of epithelial markers such as E-cadherin protein, accompanied by the upregulation of mesenchymal markers such as N-cadherin and Vimentin^22^. Previous animal experiments have shown that the overexpression of SOCS2 can inhibit STAT5 activation and thus suppress epithelial-mesenchymal transition in lung adenocarcinoma^23^. Through GSEA, we found that SOCS2 can affect epithelial mesenchymal transformation in hepatoblastoma. To validate the results of bioinformatics analysis, we conducted interference and overexpression experiments on HB cells. Western blot results showed that overexpression of SOCS2 in HB cells led to an increase in E-cadherin expression, while the expression of N-cadherin, Vimentin, and Snail proteins, which represent the mesenchymal phenotype, decreased. SOCS2 can inhibit EMT in HB cell lines, further demonstrating its ability to suppress the invasion and metastasis of hepatoblastoma.

## Conclusion

Our study found low expression of SOCS2 in hepatoblastoma tissues and cells. SOCS2 is associated with hepatoblastoma vascular invasion and tumor metastasis. Overexpression of SOCS2 can inhibit the migration, invasion, and EMT of HB cells. SOCS2 may inhibit the migration and invasion of HB cells by inhibiting the JAK2/STAT5 signaling pathway. In conclusion, our study reports for the first time the significance of the SOCS2/JAK2/STAT5 axis in hepatoblastoma metastasis (Supplementary Information S1).

### Supplementary Information

Supplementary Figures.

## Data Availability

The datasets presented in this study can be found in online repositories. The names of the repository/repositories and accession number(s) can be found below: [GSE131329 AND https://www.ncbi.nlm.nih.gov/geo/query/acc.cgi?acc=GSE131329]. Other data and materials used during the current study are available from the corresponding author on reasonable request.

## References

[1] Kehm, R. D., Osypuk, T. L., Poynter, J. N., Vock, D. M. & Spector, L. G. Do pregnancy characteristics contribute to rising childhood cancer incidence rates in the United States? *Pediatr. Blood Cancer* **65**, 1. 10.1002/pbc.26888 (2018). 10.1002/pbc.26888 PMC5766387 29160610

[2] Johnston, M. E., 2nd *et al.* Olaparib inhibits tumor growth of hepatoblastoma in patient-derived xenograft models. *Hepatology (Baltimore, Md.)* **74**, 2201–2215. 10.1002/hep.31919 (2021). 10.1002/hep.31919 PMC8463483 34037269

[3] Gong W, Han Z, Fang F, Chen L (2022). Yap expression is closely related to tumor angiogenesis and poor prognosis in hepatoblastoma. Fetal Pediatr. Pathol..

[4] Liu W, Li L, Ye H, Tu W (2017). Weighted gene co-expression network analysis in biomedicine research. Sheng Wu Gong Cheng Xue Bao.

[5] Zhou XH (2022). Hepatocellular carcinoma-derived exosomal miRNA-761 regulates the tumor microenvironment by targeting the SOCS2/JAK2/STAT3 pathway. World J. Emerg. Med..

[6] Letellier E, Haan S (2016). SOCS2: Physiological and pathological functions. Front. Biosci. (Elite edition).

[7] Li B (2017). Development and validation of a three-gene prognostic signature for patients with hepatocellular carcinoma. Sci. Rep..

[8] Dai W (2022). LPIN1 is a regulatory factor associated with immune response and inflammation in sepsis. Front. Immunol..

[9] Langfelder P, Horvath S (2008). WGCNA: An R package for weighted correlation network analysis. BMC Bioinf..

[10] Cui Y (2021). ENC1 facilitates colorectal carcinoma tumorigenesis and metastasis via JAK2/STAT5/AKT axis-mediated epithelial mesenchymal transition and stemness. Front. Cell Dev. Biol..

[11] Trobaugh-Lotrario AD, Meyers RL, Feusner JH (2016). Outcomes of patients with relapsed hepatoblastoma enrolled on children's oncology group (COG) phase I and II studies. J. Pediatr. Hematol. Oncol..

[12] Qiu X (2013). Reduced expression of SOCS2 and SOCS6 in hepatocellular carcinoma correlates with aggressive tumor progression and poor prognosis. Mol. Cell. Biochem..

[13] Iglesias-Gato D (2014). SOCS2 mediates the cross talk between androgen and growth hormone signaling in prostate cancer. Carcinogenesis.

[14] Haffner MC (2007). Favorable prognostic value of SOCS2 and IGF-I in breast cancer. BMC Cancer.

[15] Miller ME (2004). Suppressor of cytokine signaling-2: A growth hormone-inducible inhibitor of intestinal epithelial cell proliferation. Gastroenterology.

[16] Wernig G (2008). Efficacy of TG101348, a selective JAK2 inhibitor, in treatment of a murine model of JAK2V617F-induced polycythemia vera. Cancer Cell.

[17] Wu L (2022). CXCL9 influences the tumor immune microenvironment by stimulating JAK/STAT pathway in triple-negative breast cancer. Cancer Immunol. Immunother..

[18] Masuzaki R (2016). SOCS2 balances metabolic and restorative requirements during liver regeneration. J. Biol. Chem..

[19] Kaltenecker D (2019). Hepatic growth hormone—JAK2–STAT5 signalling: Metabolic function, non-alcoholic fatty liver disease and hepatocellular carcinoma progression. Cytokine.

[20] Wang M (2020). Intracellular matrix Gla protein promotes tumor progression by activating JAK2/STAT5 signaling in gastric cancer. Mol. Oncol..

[21] Kinslechner K (2018). Malignant phenotypes in metastatic melanoma are governed by SR-BI and its association with glycosylation and STAT5 activation. Mol. Cancer Res..

[22] Huang Z, Zhang Z, Zhou C, Liu L, Huang C (2022). Epithelial-mesenchymal transition: The history, regulatory mechanism, and cancer therapeutic opportunities. MedComm.

[23] Zhou Y (2018). Suppressor of cytokine signalling-2 limits IGF1R-mediated regulation of epithelial-mesenchymal transition in lung adenocarcinoma. Cell Death Dis..

