# Supplementary material for: SOCS2 inhibits hepatoblastoma metastasis via downregulation of the JAK2/STAT5 signal pathway
Source: Sci Rep. 2023 Dec 9;13:21814. doi: 10.1038/s41598-023-48591-7 (PMC10710468; doi:10.1038/s41598-023-48591-7)

Figure2B

β-actin

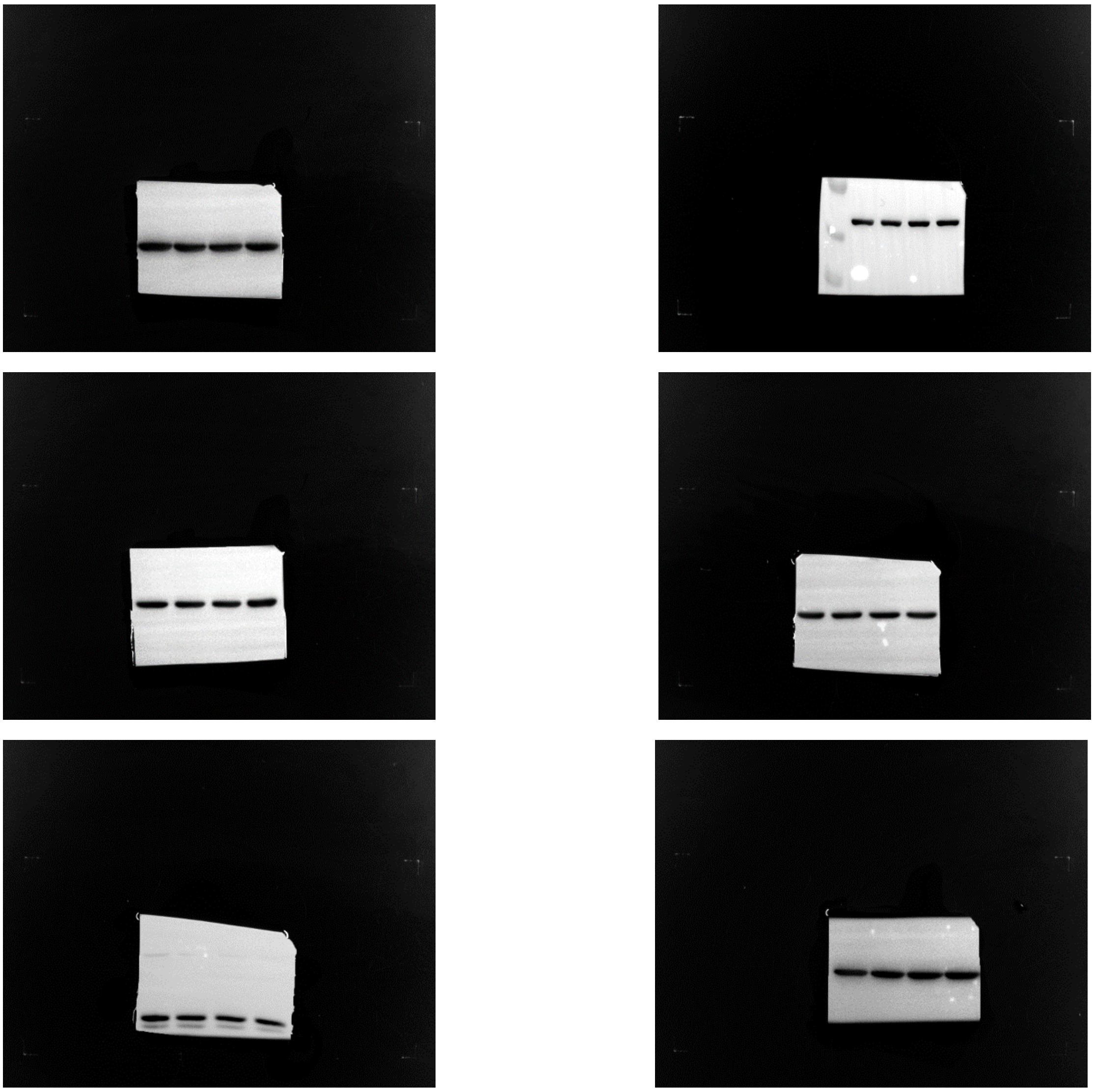

SOCS2

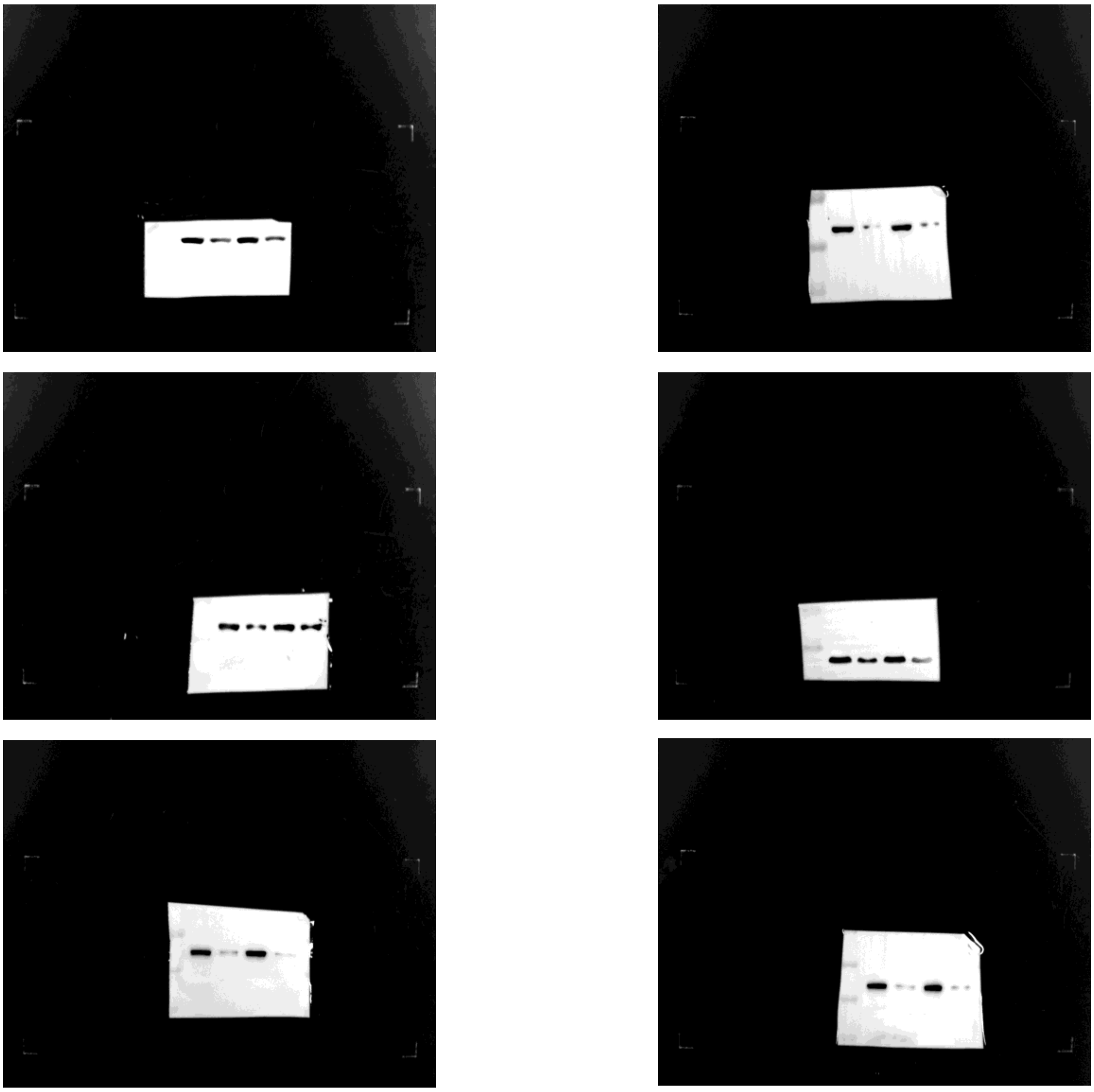

Figure5A

Huh6

JAK2

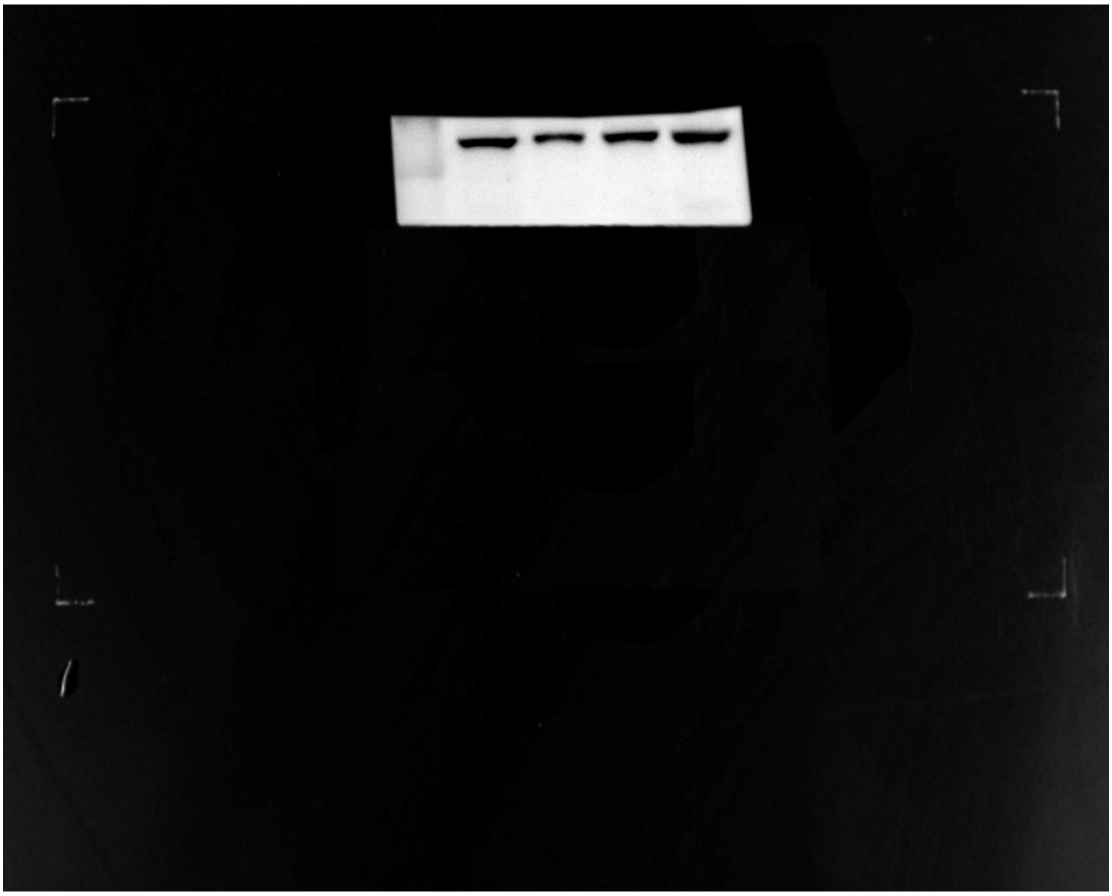

Figure5A

Huh6

p-JAK2

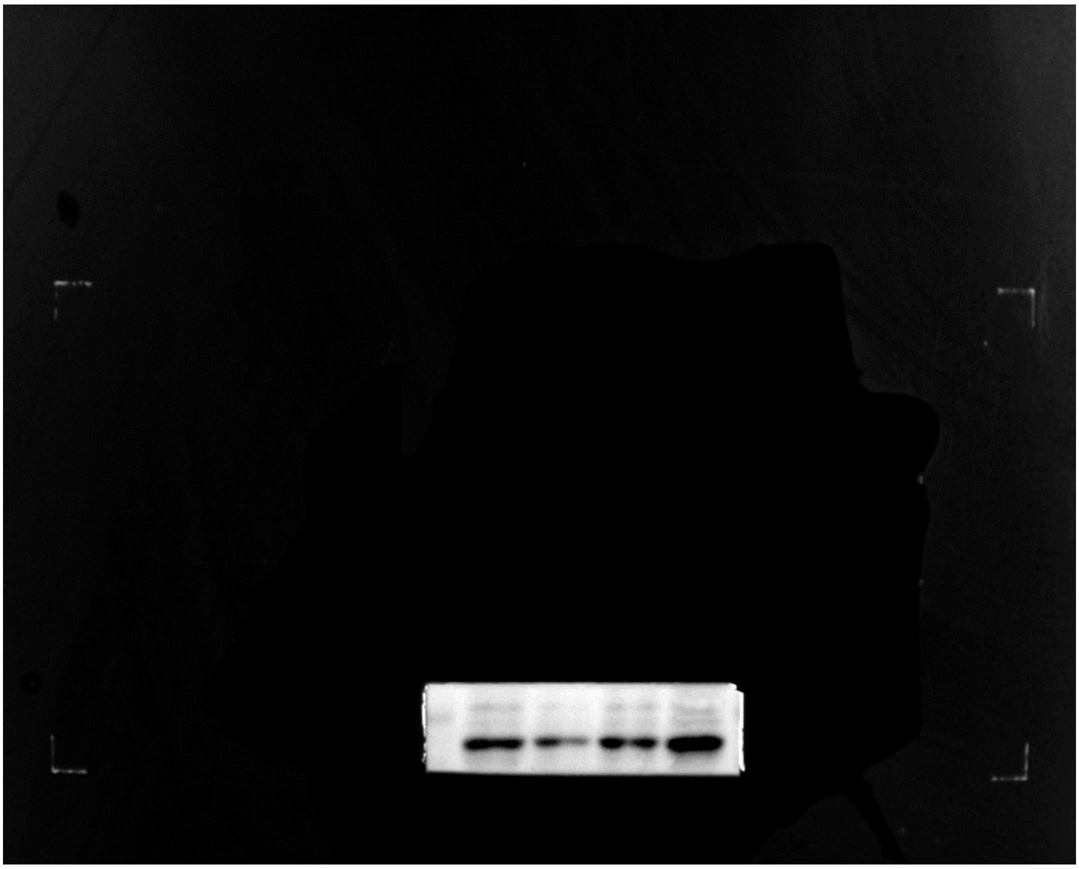

Figure5A

Huh6

STAT5

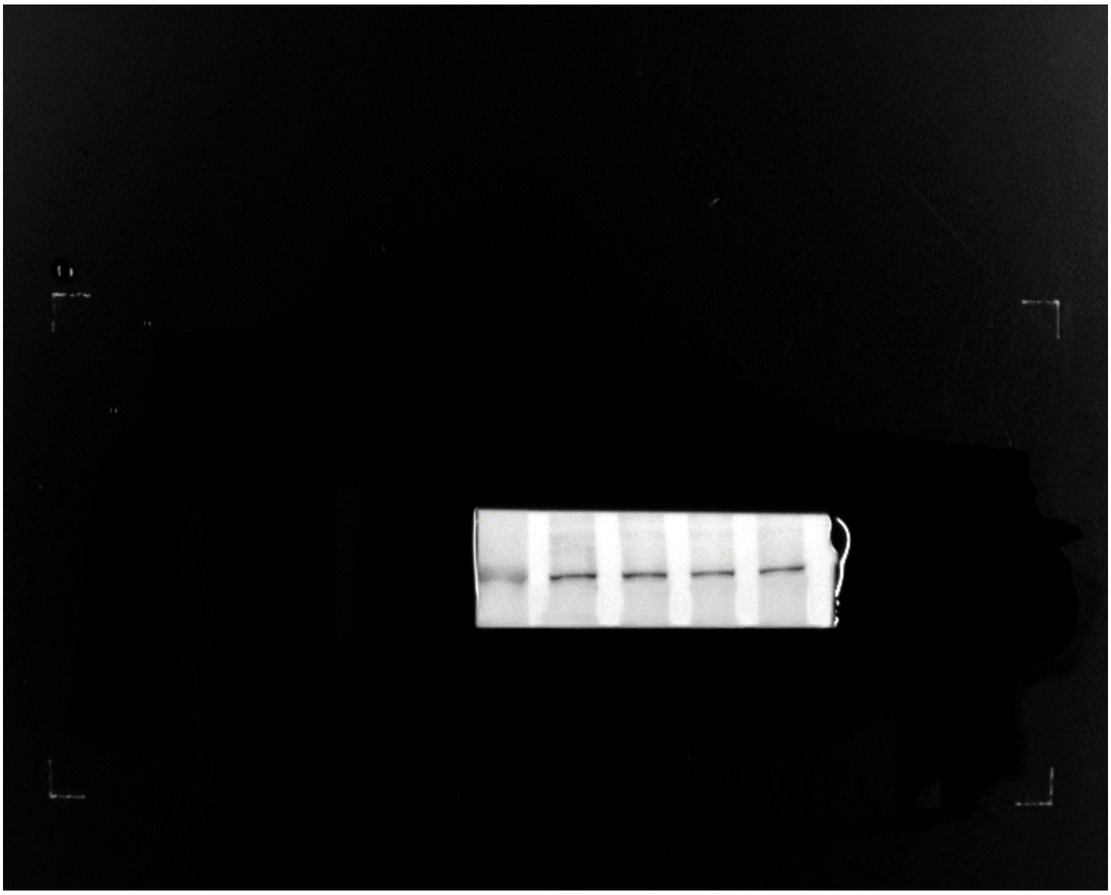

Figure5A
Huh6
p-STAT5

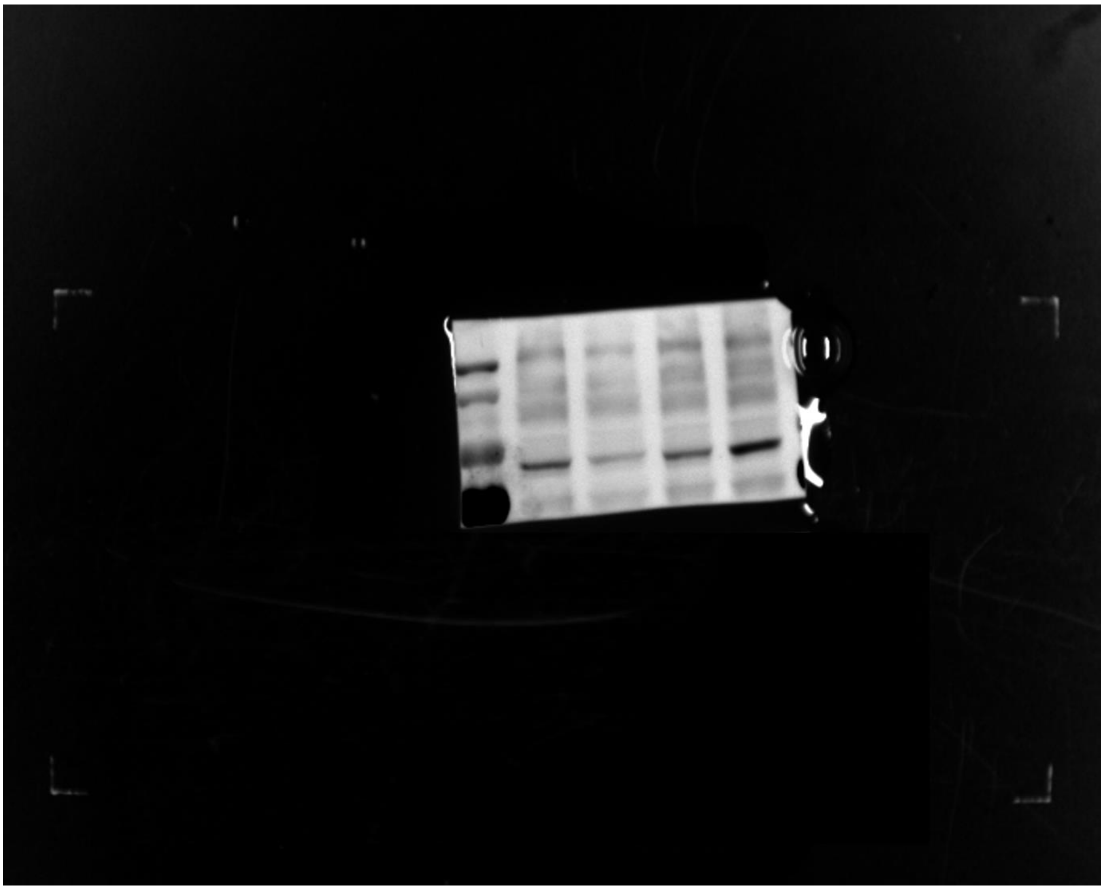

Figure5A
Huh6
SOCS2

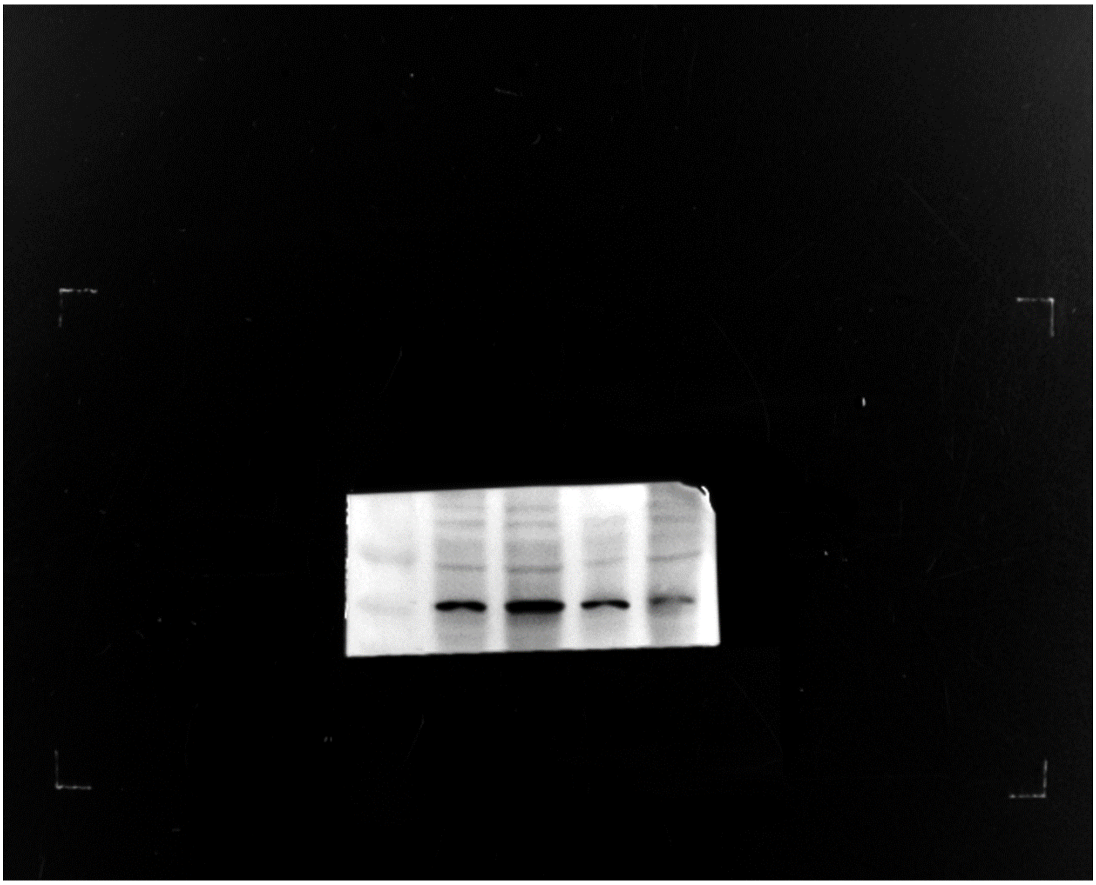

Figure5A

Huh6

β-actin

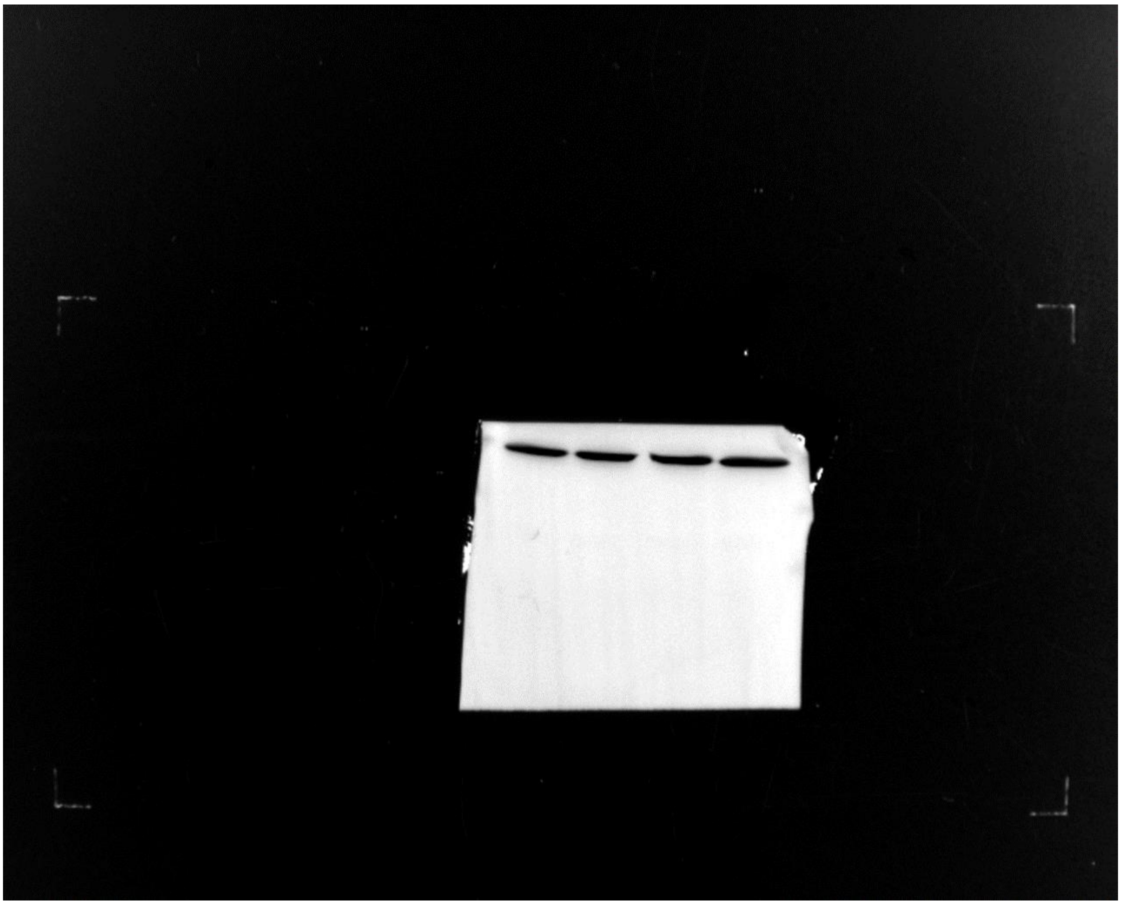

Figure5A

HepG2

JAK2

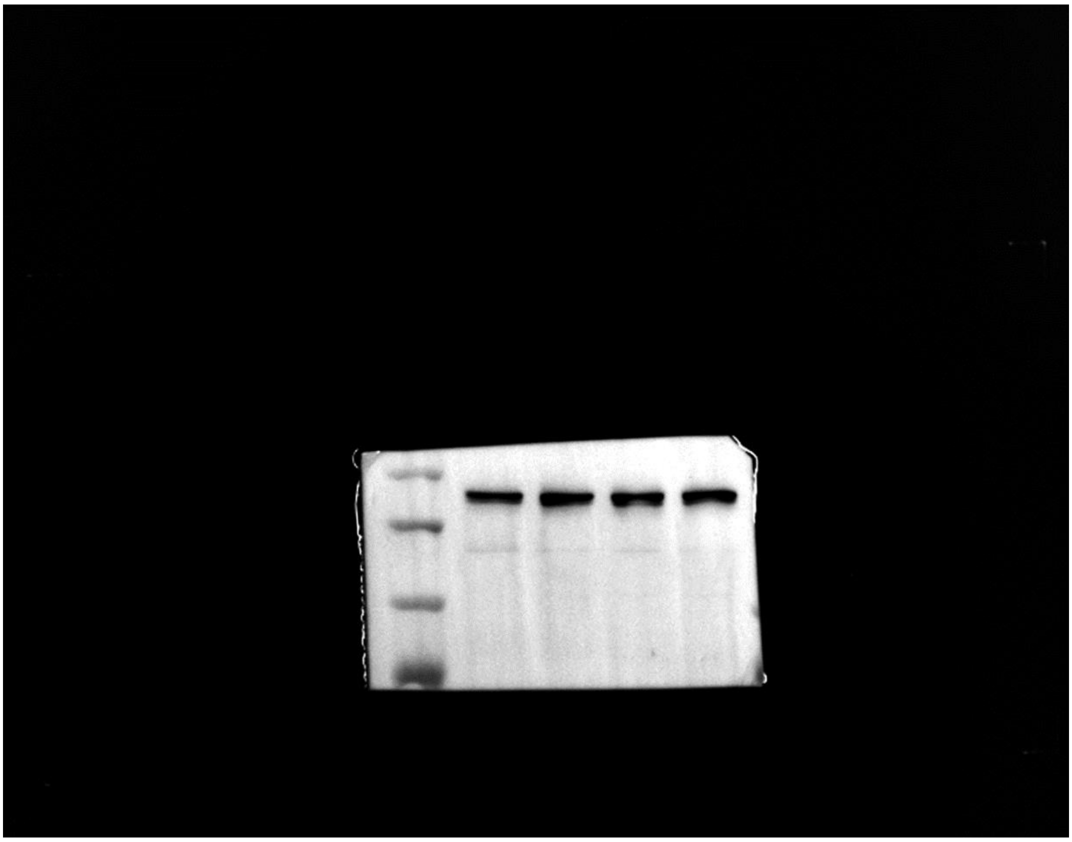

Figure5A

HepG2

p-JAK2

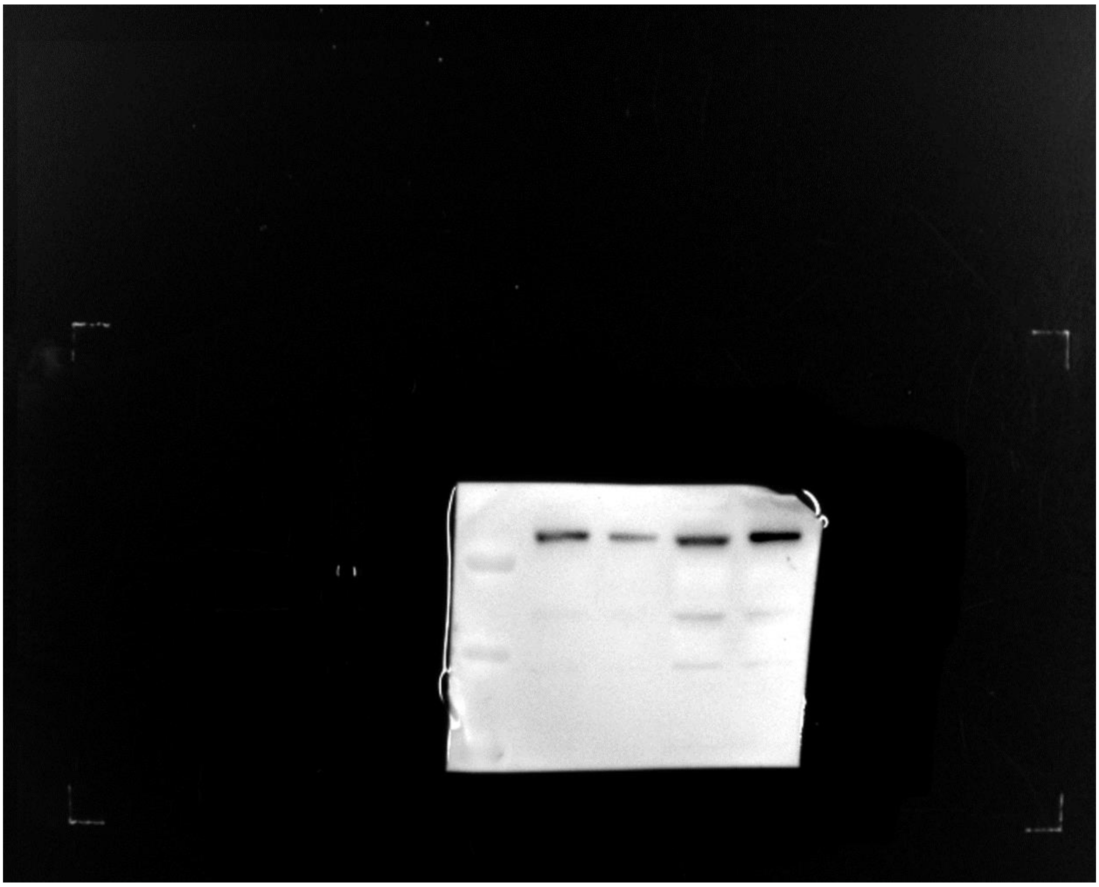

Figure5A

HepG2

STAT5

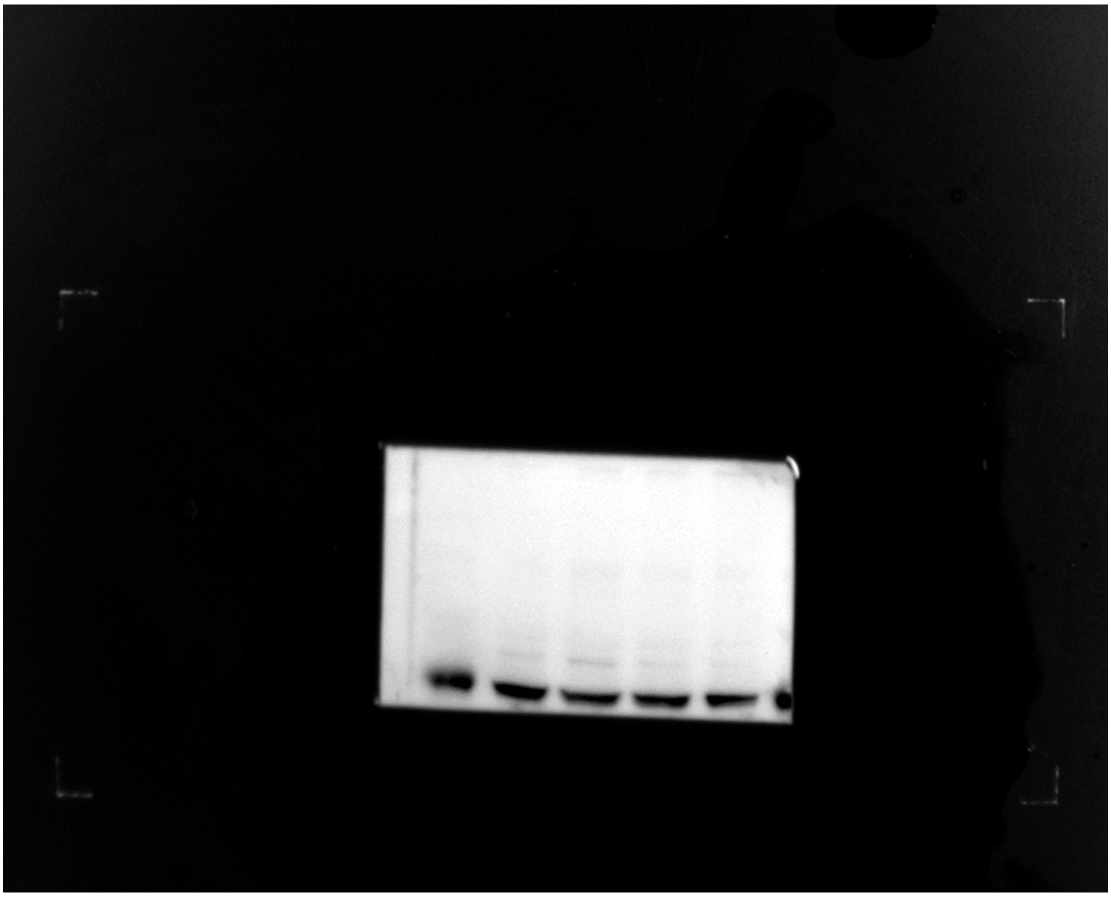

Figure5A

HepG2

p-STAT5

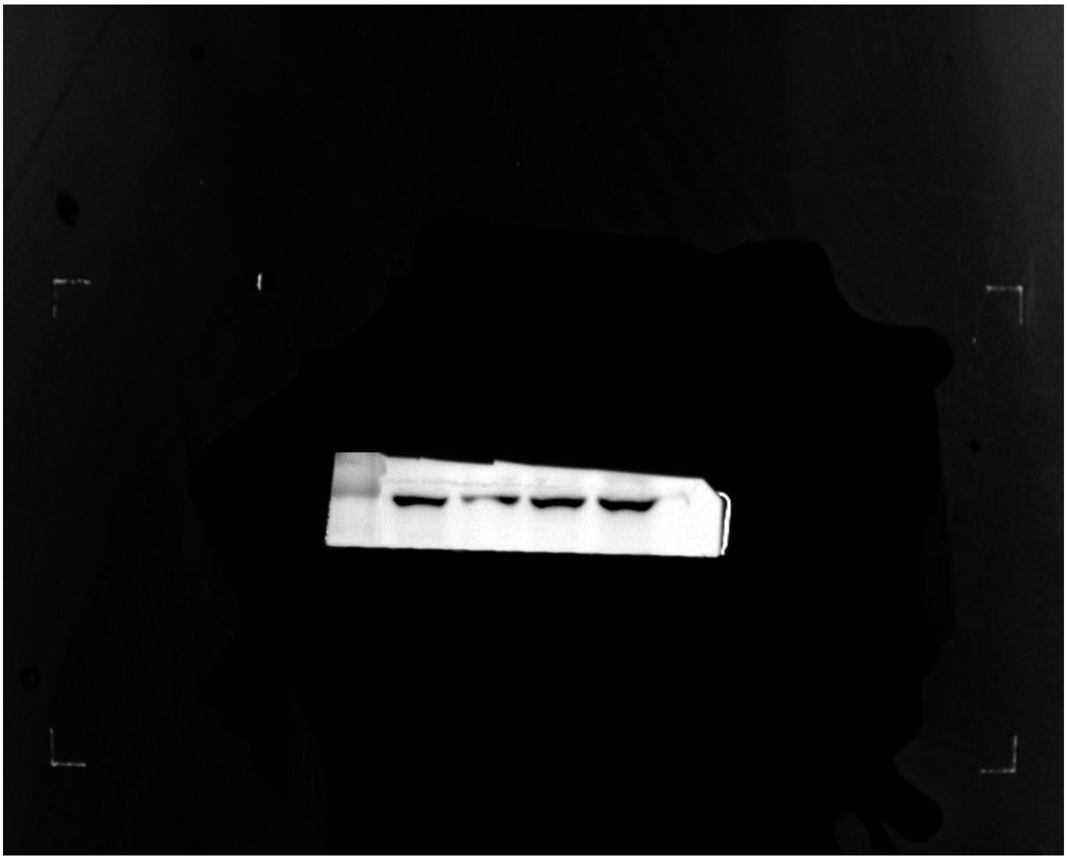

Figure5A

HepG2

SOCS2

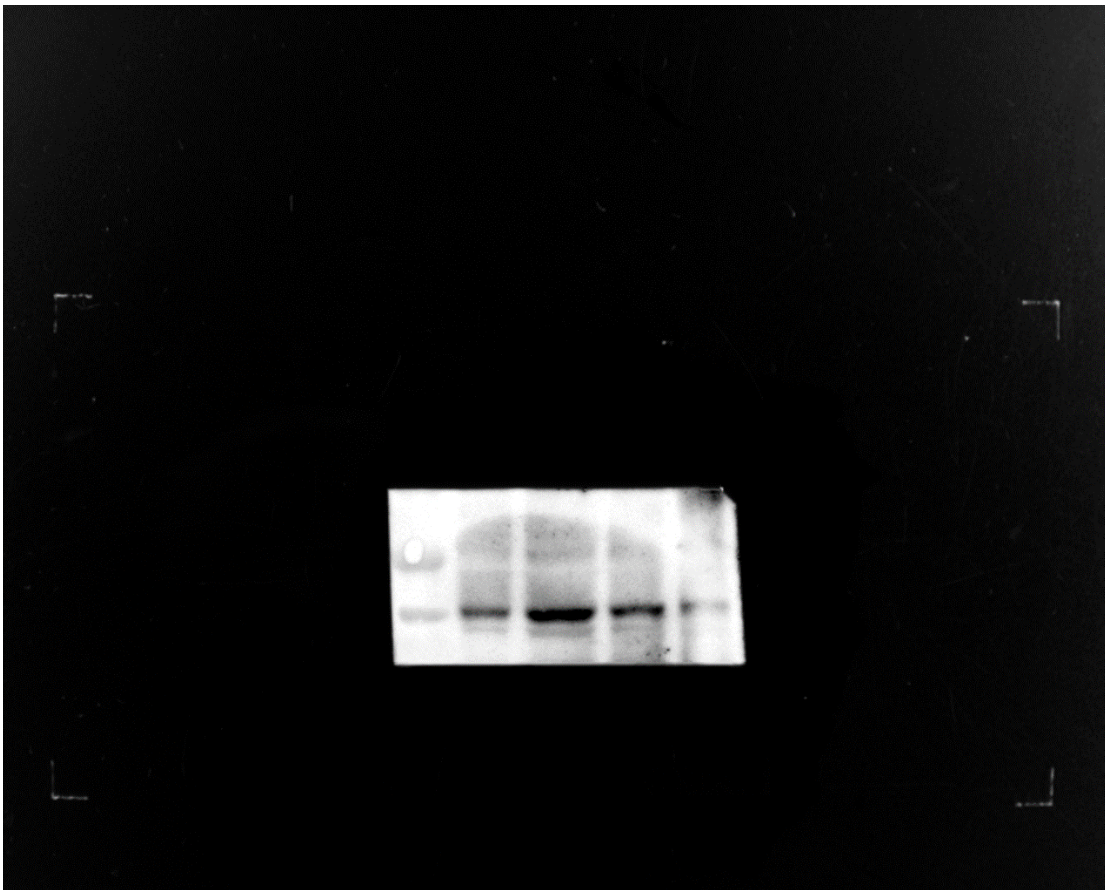

Figure5A

HepG2

ACTB

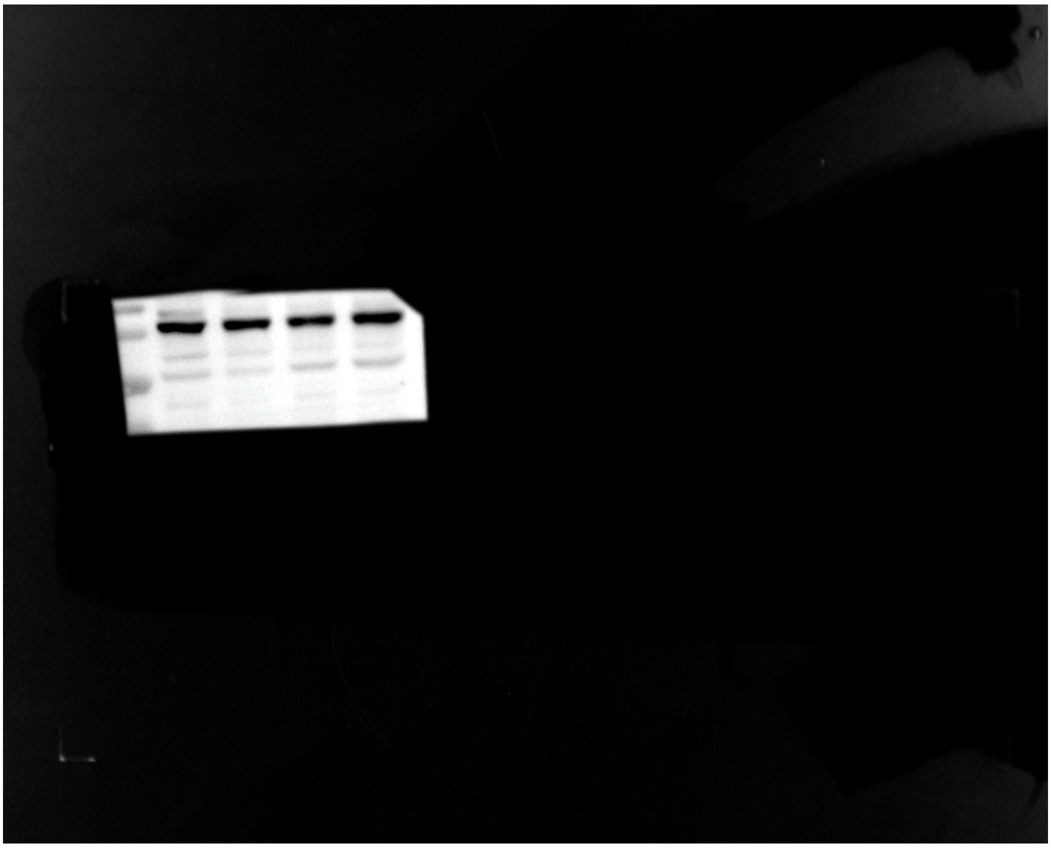

Figure5B

HepG2

E-ca

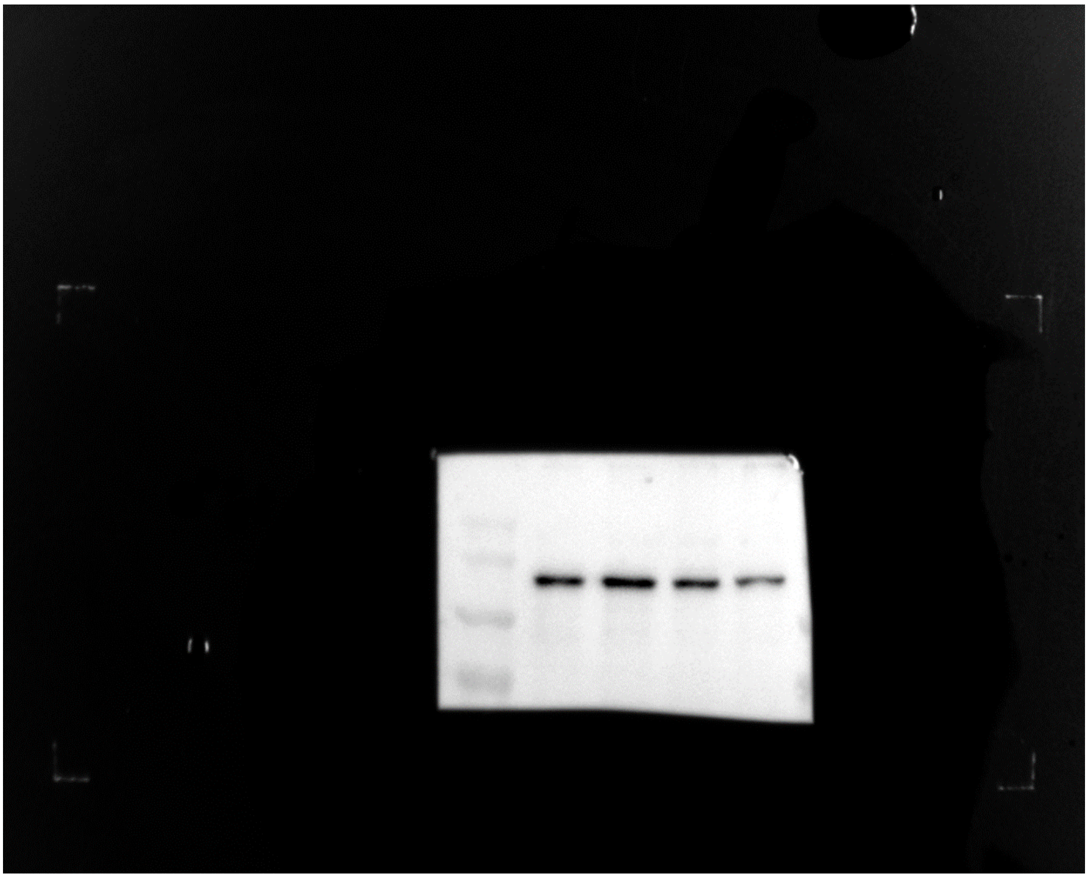

Figure5B

HepG2

N-ca

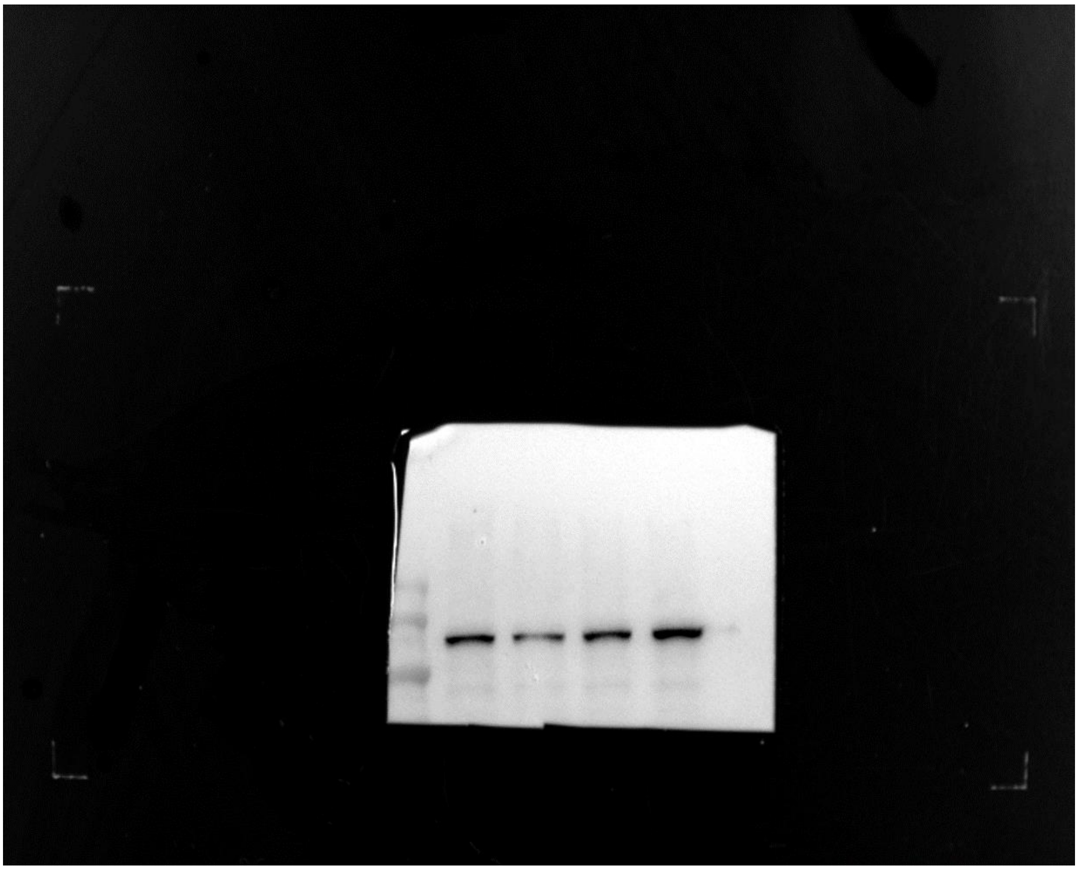

Figure5B
HepG2
Vimentin
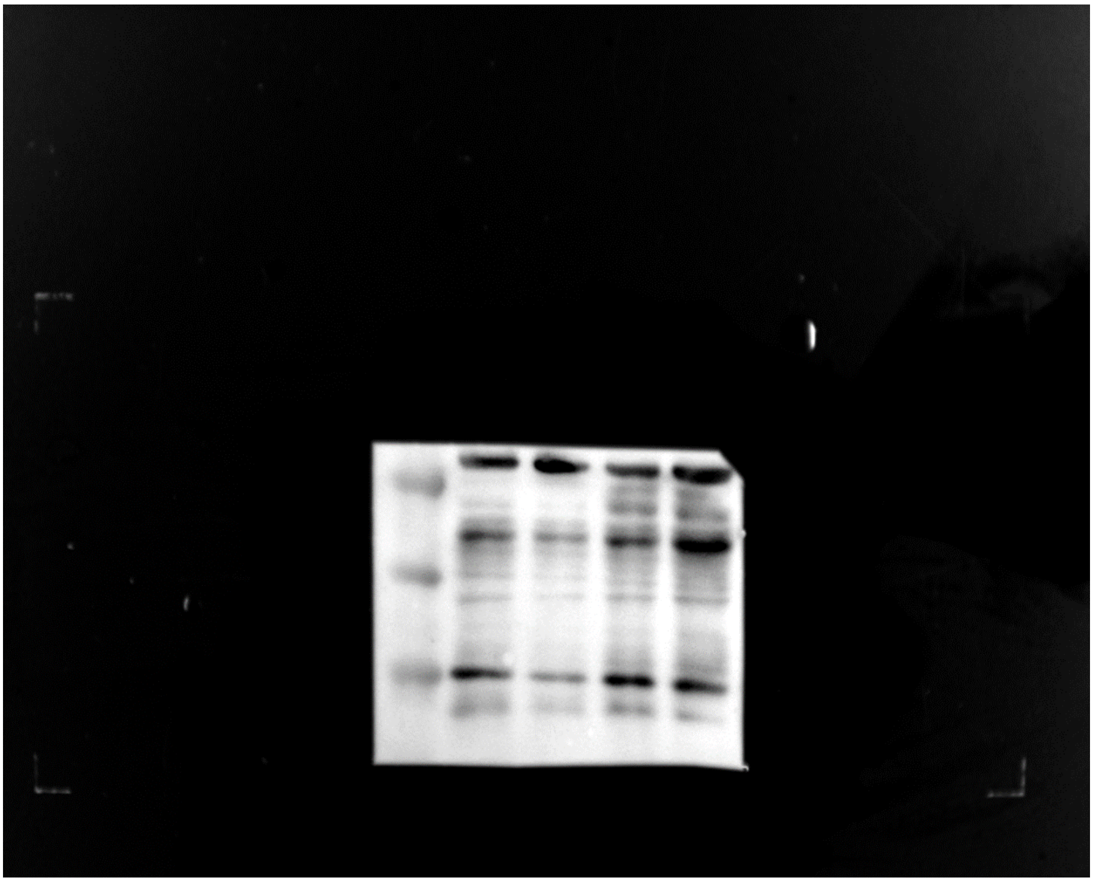

Figure5B

HepG2

Snail

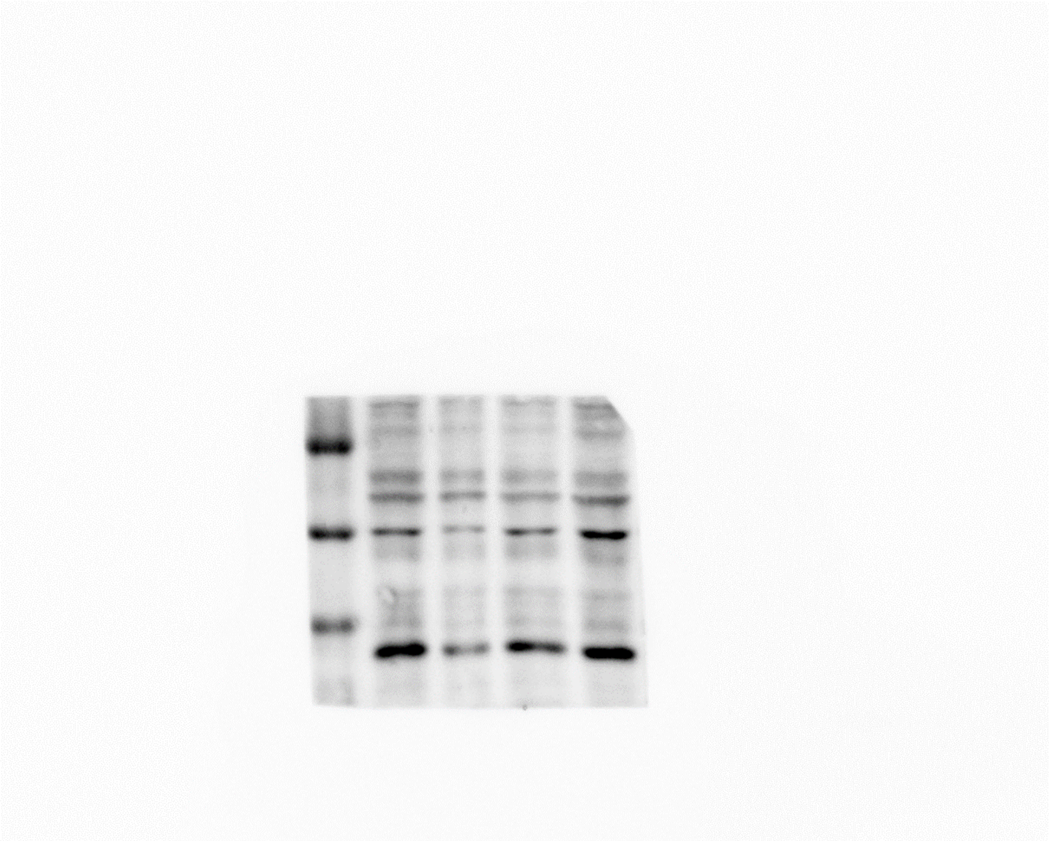

Figure5B

HepG2

SOCS2

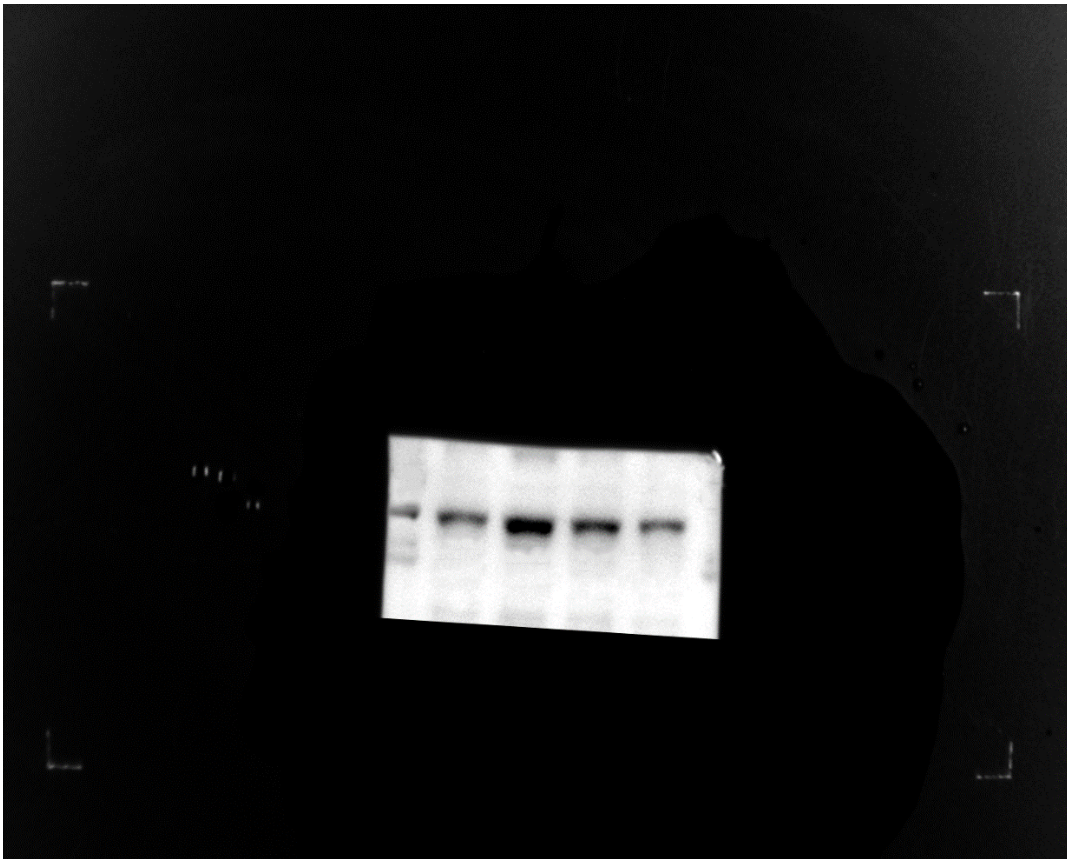

Figure5B

HepG2

actb

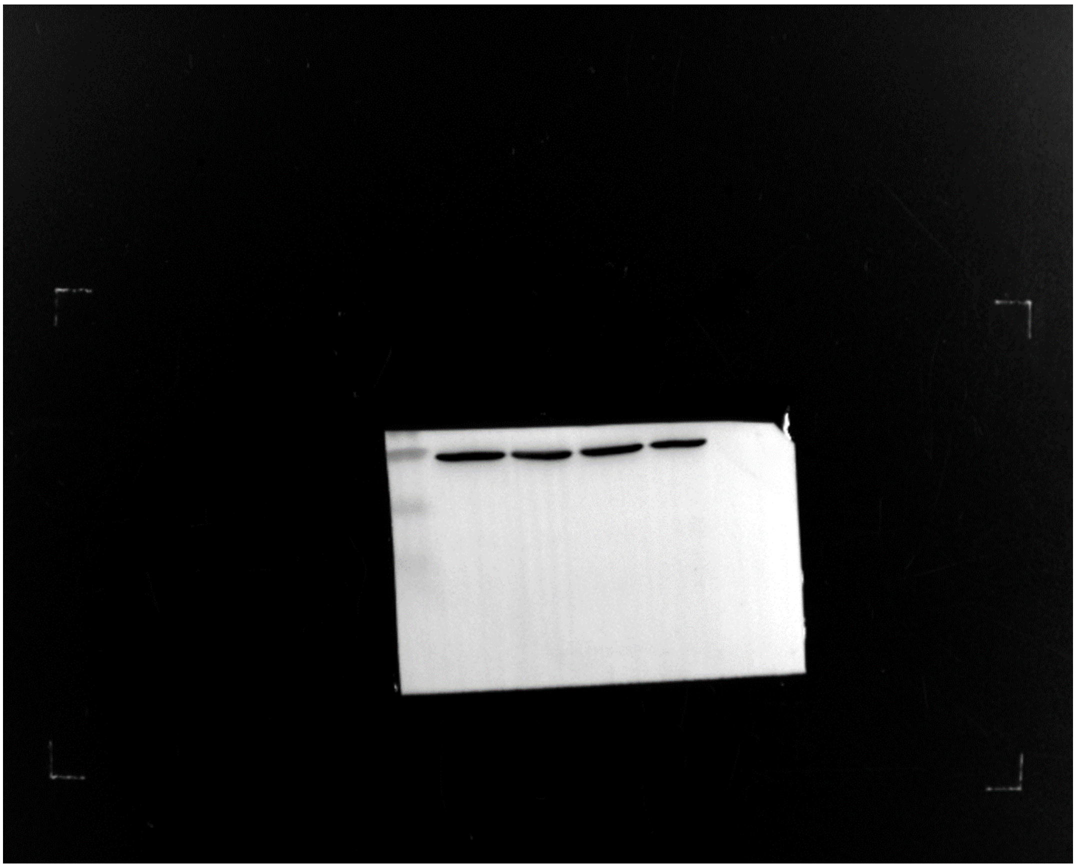

Figure5B

Huh6

E-ca

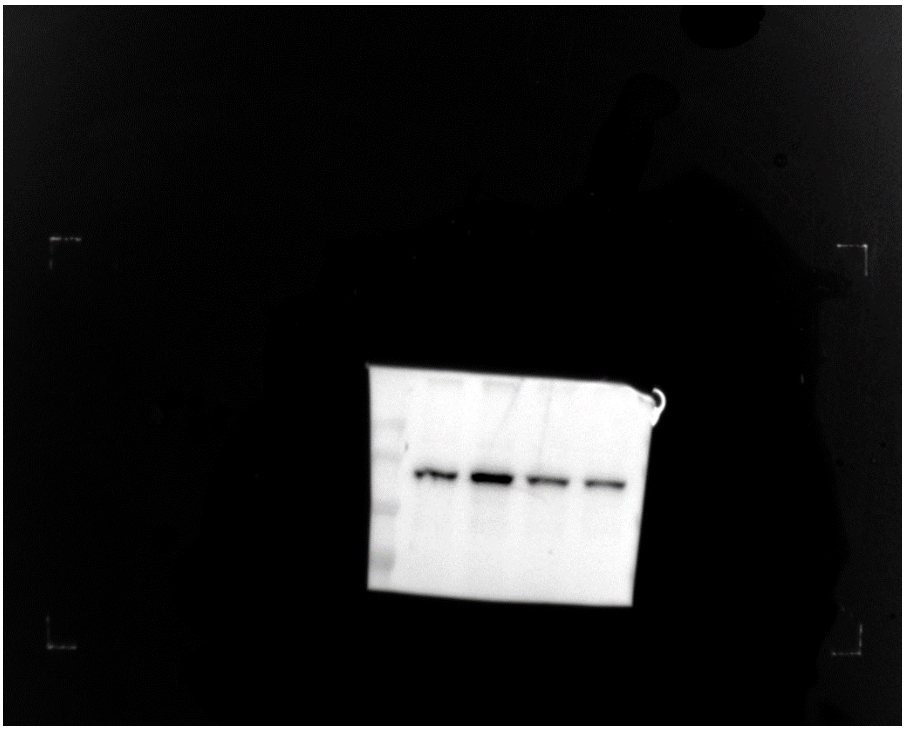

Figure5B

Huh6

N-ca

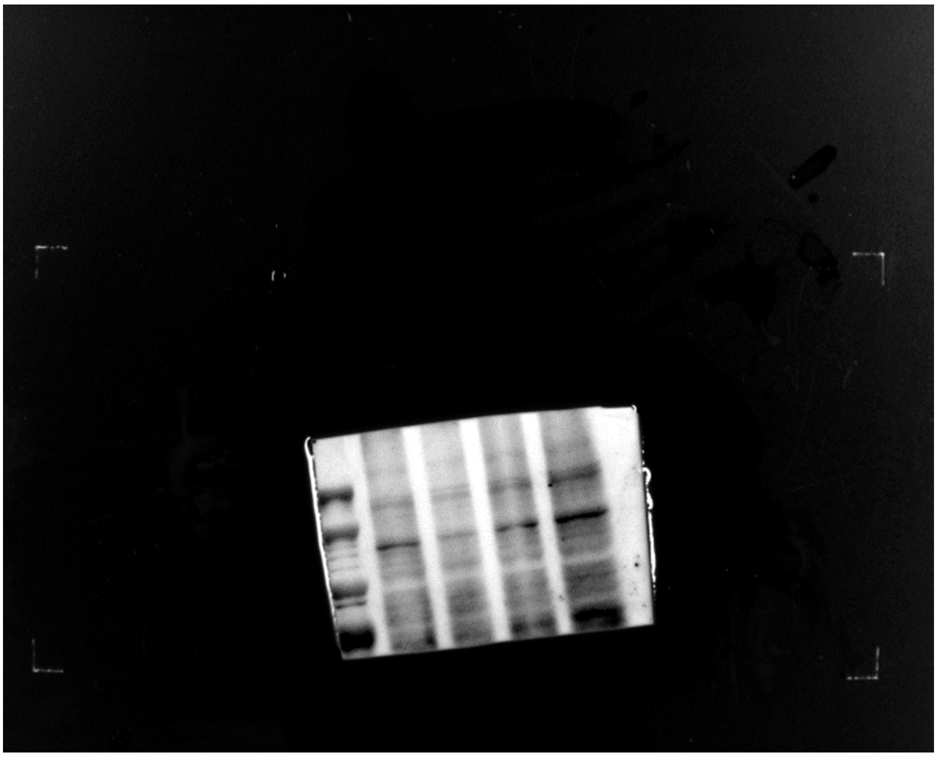

Figure5B

Huh6

Vimentin

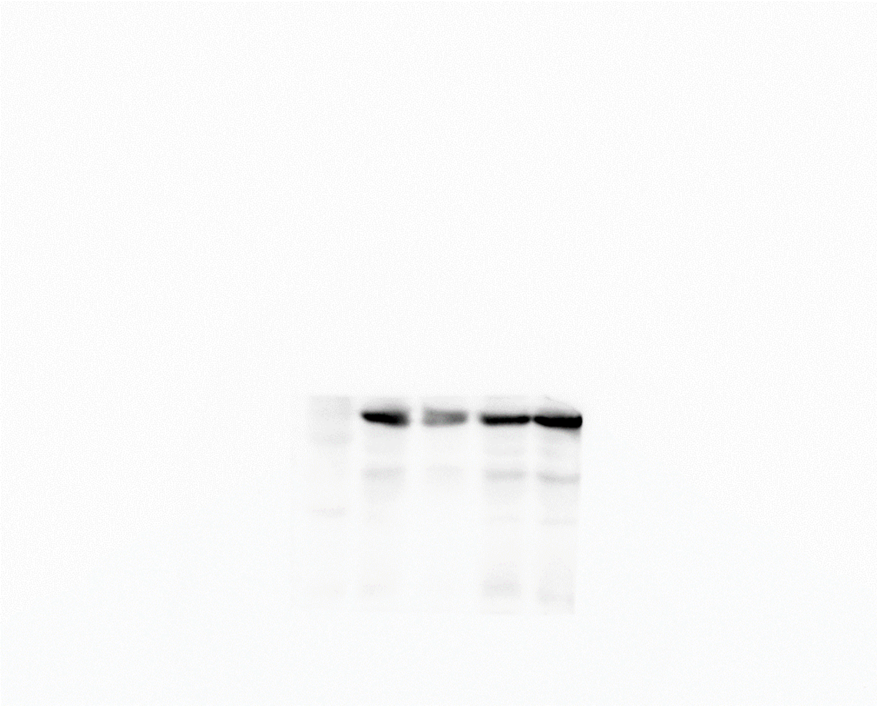

Figure5B
Huh6
Snail

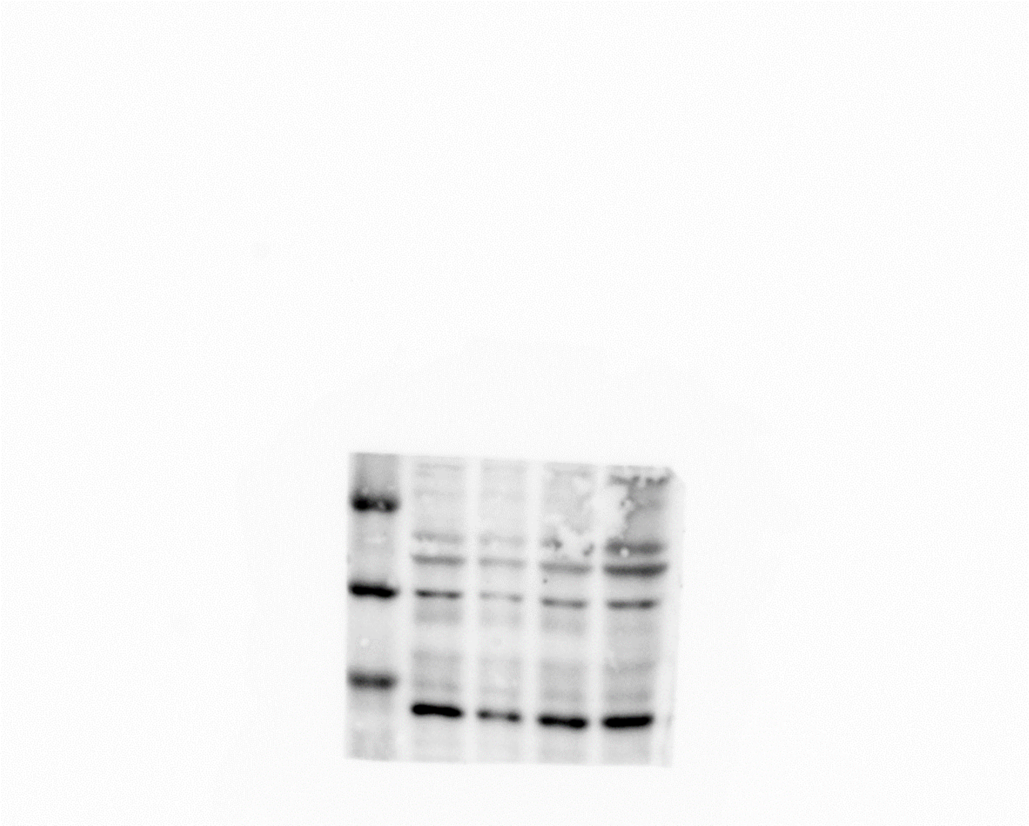

Figure5B
Huh6
SOCS2

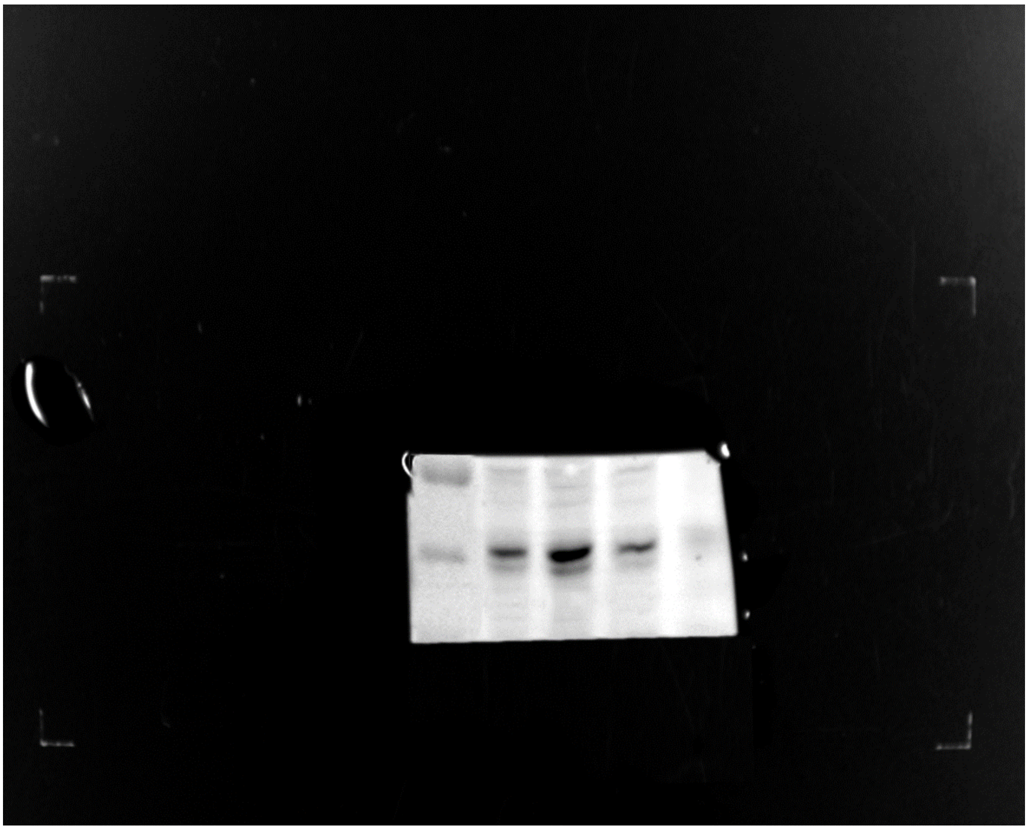

Figure5B
Huh6
actb

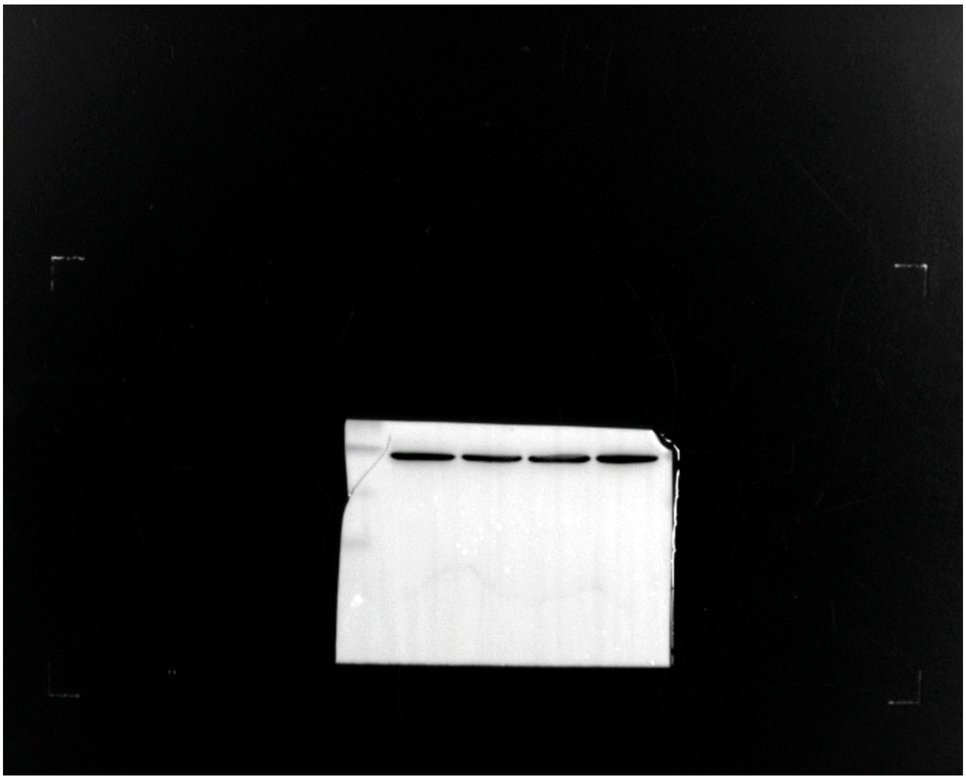

Figure6A
Huh6
JAK2

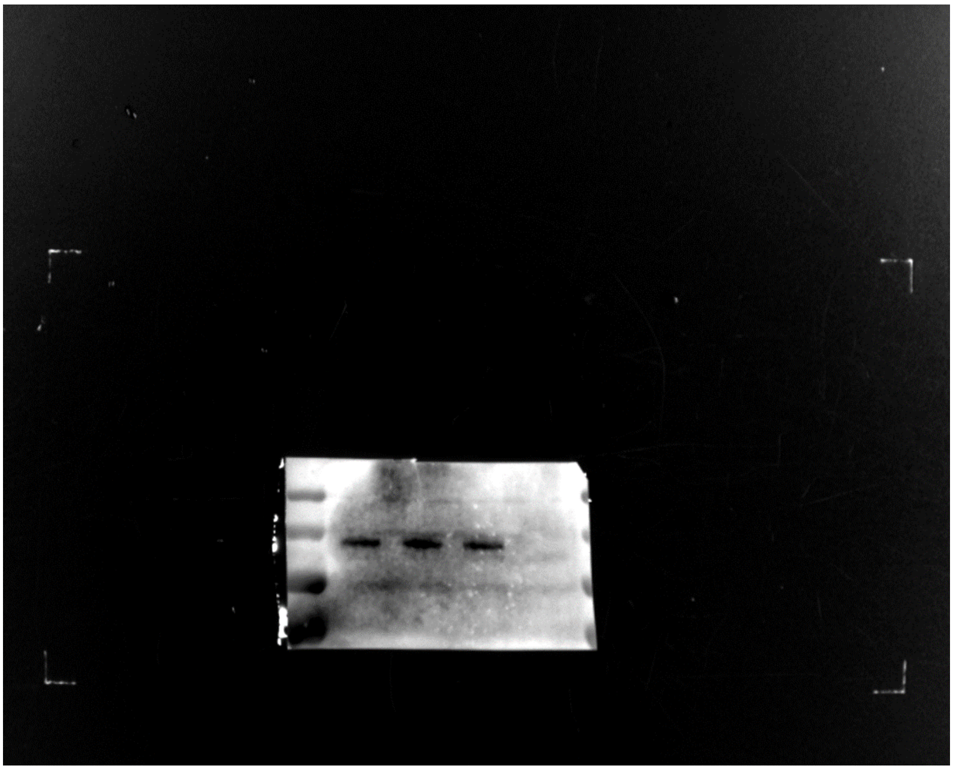

Figure6A

Huh6

p-JAK2

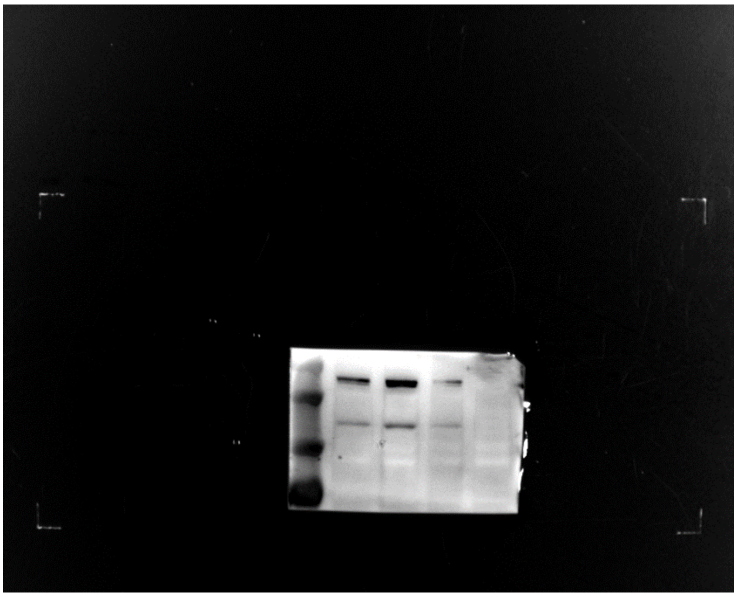

Figure6A

Huh6

STAT5

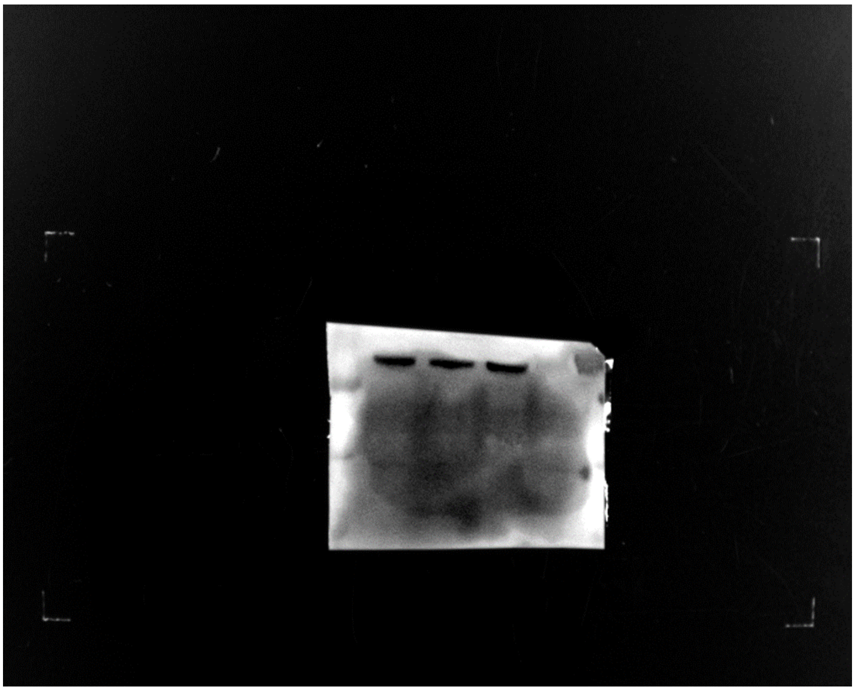

Figure6A
Huh6
p-STAT5

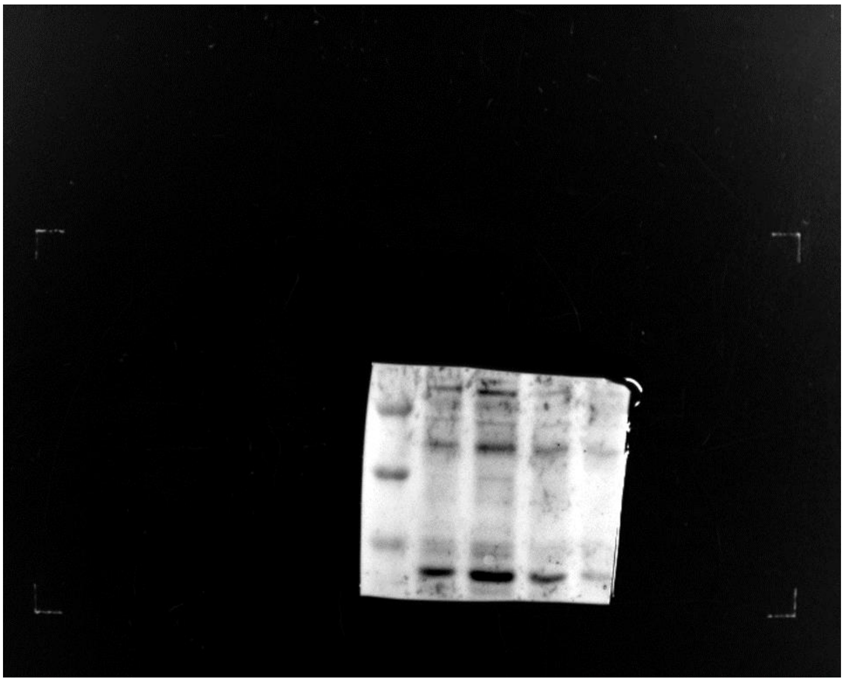

Figure6A

Huh6

Actb

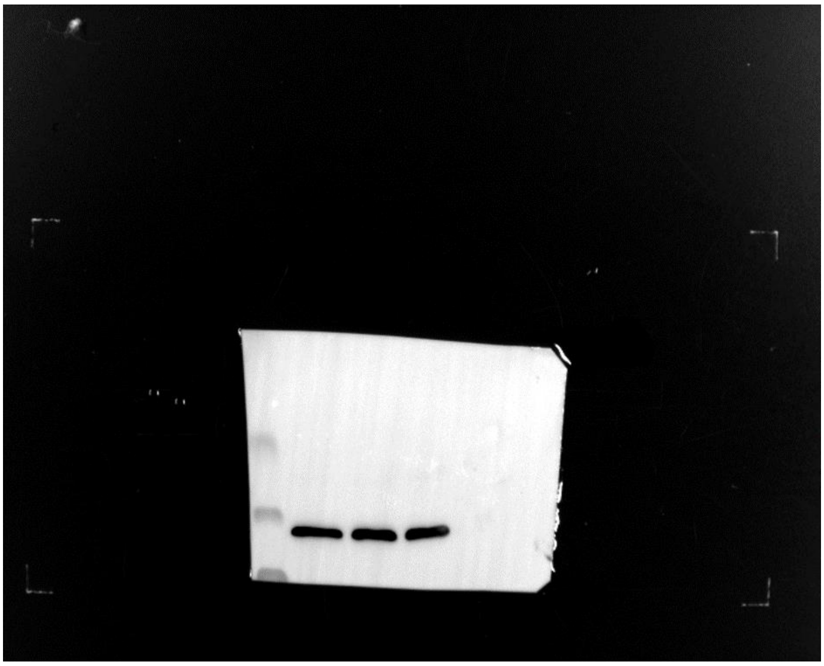

Figure6B

Huh6

E-ca

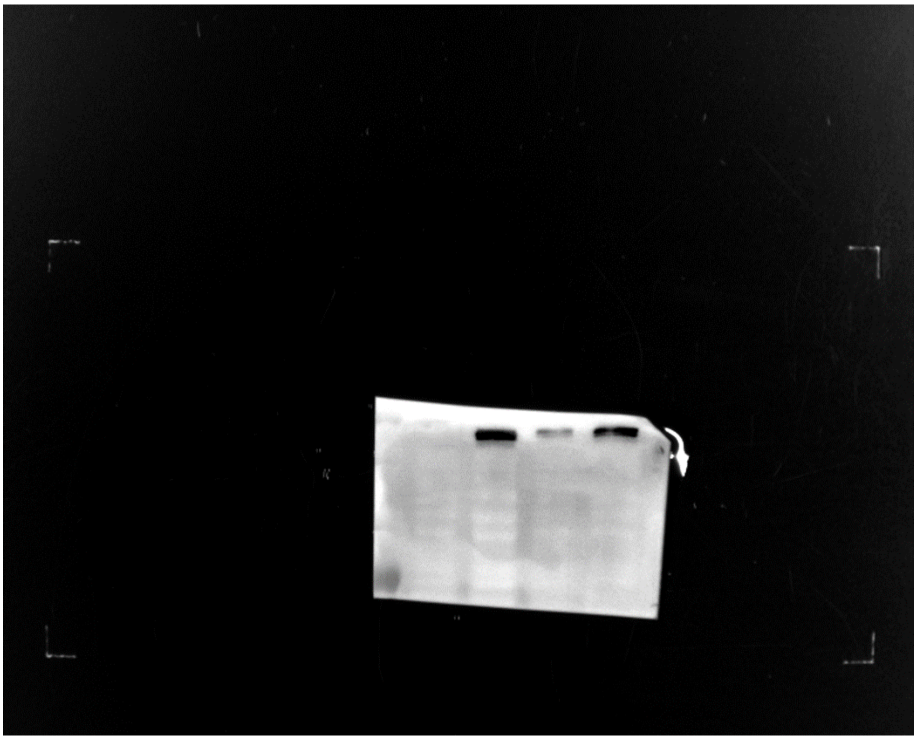

Figure6B

Huh6

N-ca

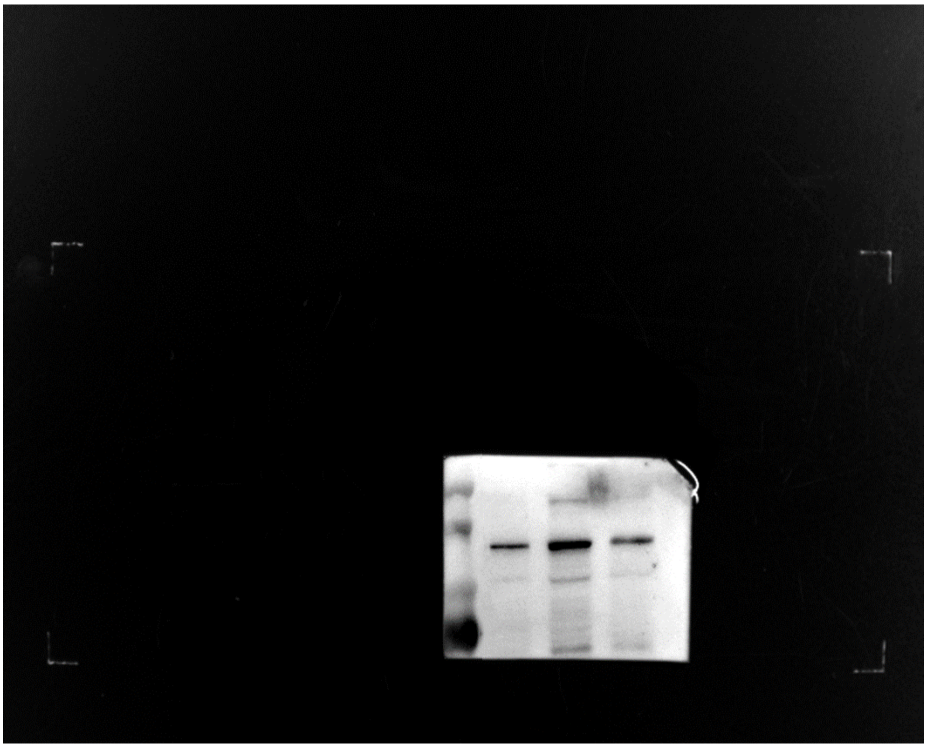

Figure6B
Huh6
Vimentin

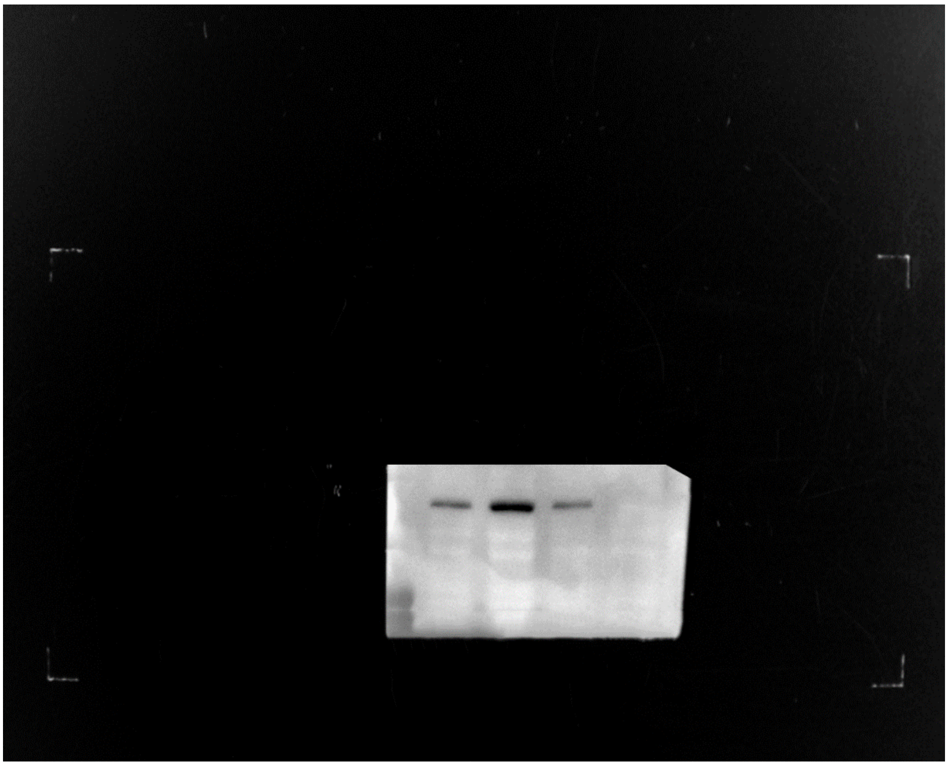

Figure6B
Huh6
Snail

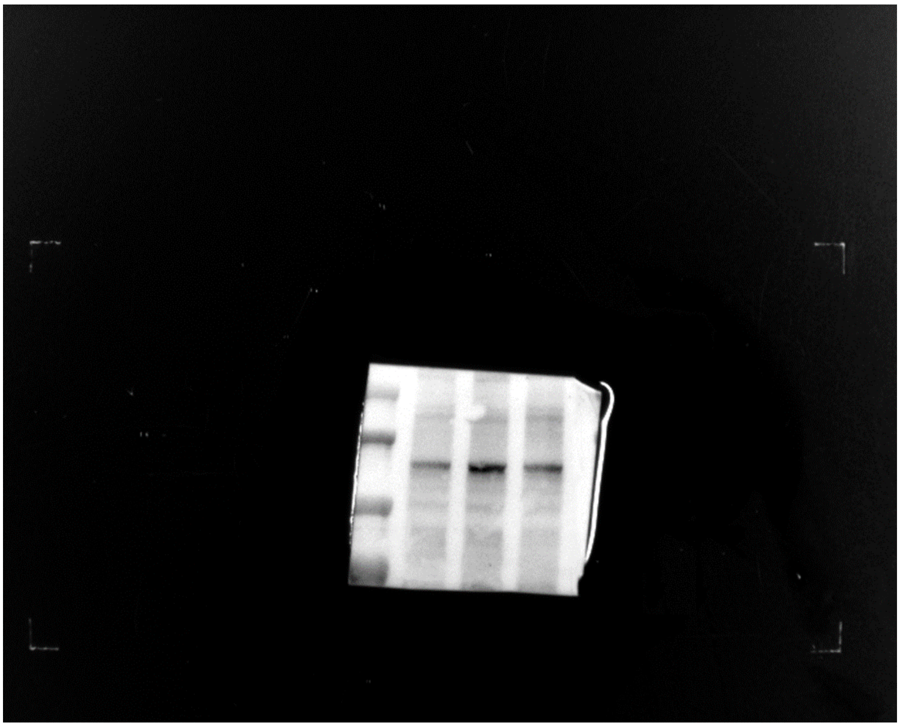

Figure6B

Huh6

Actb

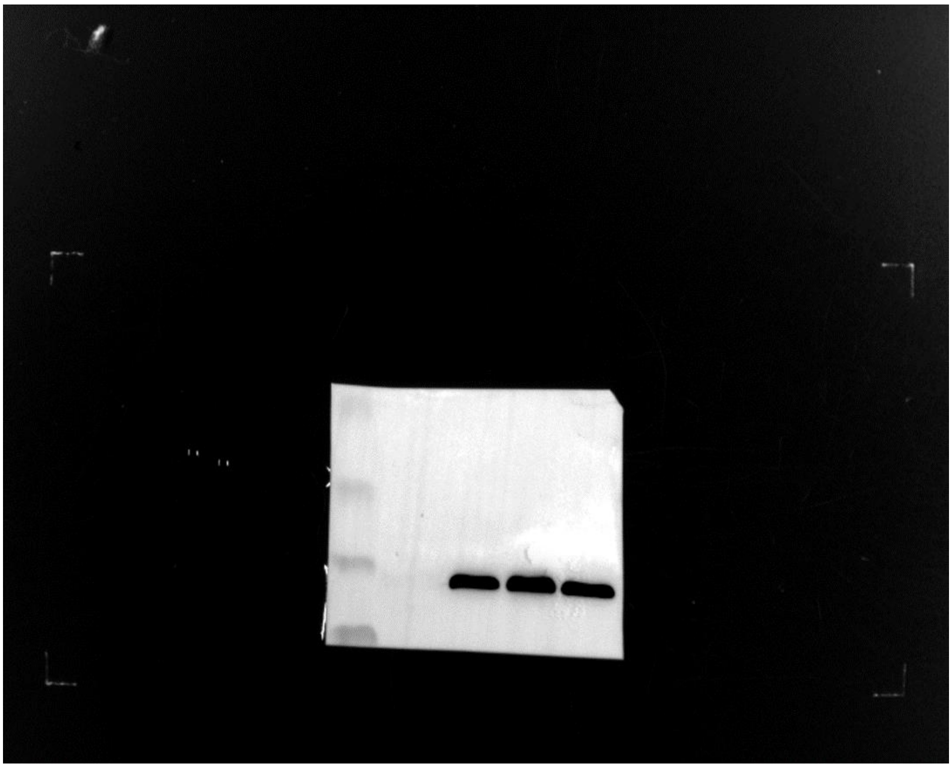

Supplement: Supplementary file 1 — Supplementary Figures. [file 41598_2023_48591_MOESM1_ESM.pdf]
